# Sleep duration in preschool age and later behavioral and cognitive outcomes: an individual participant data meta-analysis in five European cohorts

**DOI:** 10.1007/s00787-023-02149-0

**Published:** 2023-02-07

**Authors:** Kathrin Guerlich, Demetris Avraam, Tim Cadman, Lucinda Calas, Marie-Aline Charles, Ahmed Elhakeem, Silvia Fernández-Barrés, Mònica Guxens, Barbara Heude, Jesús Ibarluzea, Hazel Inskip, Jordi Julvez, Deborah A. Lawlor, Mario Murcia, Theodosia Salika, Jordi Sunyer, Muriel Tafflet, Berthold Koletzko, Veit Grote, Sabine Plancoulaine

**Affiliations:** 1 Division of Metabolic and Nutritional Medicine, Department of Pediatrics, Dr. von Hauner Children’s Hospital, LMU University Hospital Munich, Lindwurmstr. 4, 80337 Munich, Germany; 2 Université Paris Cité and Université Sorbonne Paris Nord, Inserm, INRAE, Center for Research in Epidemiology and StatisticS (CRESS), 75004 Paris, France; 3 Ined, Inserm, Joint unit Elfe, Aubervilliers, France; 4 Population Health Sciences Institute, Newcastle University, Newcastle, UK; 5 Department of Public Health, Faculty of Health and Medical Sciences, University of Copenhagen, Copenhagen, Denmark; 6 Population Health Science, Bristol Medical School, University of Bristol, Bristol, UK; 7 MRC Integrative Epidemiology Unit at the University of Bristol, Bristol, UK; 8 Barcelona Institute for Global Health (ISGlobal), Barcelona, Catalonia Spain; 9 CIBER Epidemiología y Salud Pública (CIBERESP), Instituto de Salud Carlos III, Madrid, Spain; 10 Universitat Pompeu Fabra, Barcelona, Catalonia Spain; 11 Department of Child and Adolescent Psychiatry/Psychology, Erasmus MC, University Medical Centre, Rotterdam, The Netherlands; 12 Biodonostia Health Research Institute, Group of Environmental Epidemiology and Child Development, 20014 San Sebastian, Spain; 13 Ministry of Health of the Basque Government, Sub-Directorate for Public Health and Addictions of Gipuzkoa, 20013 San Sebastian, Spain; 14 Faculty of Psychology of the University of the Basque Country, 20018 San Sebastian, Spain; 15 MRC Lifecourse Epidemiology Centre, University of Southampton, Southampton General Hospital, Southampton, UK; 16 NIHR Southampton Biomedical Research Centre, University of Southampton and University Hospital Southampton NHS Foundation Trust, Southampton, UK; 17 Clinical and Epidemiological Neuroscience Group (NeuroÈpia), Institut d’Investigació Sanitària Pere Virgili (IISPV), Reus (Tarragona), Catalonia Spain; 18 Epidemiology and Environmental Health Joint Research Unit, FISABIO–Universitat Jaume I–Universitat de València, Valencia, Spain; 19 Servicio de Análisis de Sistemas de Información Sanitaria, Conselleria de Sanitat, Generalitat Valenciana, Valencia, Spain; 20 Parc de Salut Mar, Barcelona, Catalonia Spain

**Keywords:** Preschool sleep duration, Multi-cohort analysis, Internalizing behavior, Externalizing behavior, Language, Non-verbal intelligence

## Abstract

Short sleep duration has been linked to adverse behavioral and cognitive outcomes in schoolchildren, but few studies examined this relation in preschoolers. We aimed to investigate the association between parent-reported sleep duration at 3.5 years and behavioral and cognitive outcomes at 5 years in European children. We used harmonized data from five cohorts of the European Union Child Cohort Network: ALSPAC, SWS (UK); EDEN, ELFE (France); INMA (Spain). Associations were estimated through DataSHIELD using adjusted generalized linear regression models fitted separately for each cohort and pooled with random-effects meta-analysis. Behavior was measured with the Strengths and Difficulties Questionnaire. Language and non-verbal intelligence were assessed by the Wechsler Preschool and Primary Scale of Intelligence or the McCarthy Scales of Children’s Abilities. Behavioral and cognitive analyses included 11,920 and 2981 children, respectively (34.0%/13.4% of the original sample). In meta-analysis, longer mean sleep duration per day at 3.5 years was associated with lower mean internalizing and externalizing behavior percentile scores at 5 years (adjusted mean difference: − 1.27, 95% CI [− 2.22, − 0.32] / − 2.39, 95% CI [− 3.04, − 1.75]). Sleep duration and language or non-verbal intelligence showed trends of inverse associations, however, with imprecise estimates (adjusted mean difference: − 0.28, 95% CI [− 0.83, 0.27] / − 0.42, 95% CI [− 0.99, 0.15]). This individual participant data meta-analysis suggests that longer sleep duration in preschool age may be important for children’s later behavior and highlight the need for larger samples for robust analyses of cognitive outcomes. Findings could be influenced by confounding or reverse causality and require replication.

**Supplementary Information:**

The online version contains supplementary material available at 10.1007/s00787-023-02149-0.

## Background

Healthy sleep is important for children’s physical and mental health and can have a positive influence on future health trajectories of a child [1–3]. There is growing evidence that shorter sleep duration is associated with more behavioral problems and poorer cognitive outcomes, especially in school-aged children and adolescents [4–6]. Compared with the literature in schoolchildren there is a paucity of studies in younger children of preschool age investigating this relationship [7, 8].

Early childhood is a sensitive period where both brain maturation and sleep habits are developing with continuation throughout childhood [9]. Insufficient sleep in these early years of life can have lasting impacts on a child’s development [8]. Chaput et al. [7] reported in a systematic review of 25 studies that shorter sleep duration was associated with poorer emotional regulation in children aged 0 to 4 years, while for sleep duration and cognitive development (16 studies) results were less clear. Authors concluded that the evidence was mainly based on cross-sectional studies and the high level of between-study heterogeneity made meta-analysis infeasible. Another systematic review of 26 studies on sleep and its relation to behavior and cognition in preschoolers by Reynaud et al. [8] suggested that a higher quantity and quality of sleep was associated with better behavioral outcomes and receptive vocabulary, but found no association for other cognitive outcomes. They concluded that mainly cross-sectional designs (69% of studies), incomplete adjustment for confounders, weak effect sizes and small sample sizes (< 500) limited the validity of the results. Both reviews showed that only a few studies in preschoolers have examined the relationship between sleep duration and later behavioral or cognitive outcomes. They tend to suggest negative associations between sleep duration and internalizing and externalizing problems as well as mixed results for language and non-verbal intelligence in healthy preschoolers [10–14]. With our study involving five European pregnancy and birth cohorts with available data on sleep duration and behavior and cognition, we aimed to examine these previously reported results in a larger sample of preschool aged children. The objective of our study was to investigate the associations between sleep duration in early childhood (~ 3.5 years) and later behavioral problems (internalizing and externalizing) and cognitive outcomes (language and non-verbal intelligence) in children (~ 5 years) using individual participant data.

## Methods

### Study design and study population

Our study used harmonized data from an international cross-cohort collaboration, the European Union Child Cohort Network established in the Horizon 2020 Project LifeCycle [15–17]. A cohort was eligible for our study if it had harmonized preschool sleep at 2 to 4 years of age and behavior (internalizing, externalizing) or/and cognition data (language, non-verbal intelligence) from ages 4 to 6 years. Five cohorts participated: ALSPAC (Avon Longitudinal Study of Parents and Children, United Kingdom, *n* = 4847 eligible children) [18, 19], EDEN (Étude des Déterminants pré et postnatals du développement et de la santé de l’Enfant, France, *n* = 1015 eligible children) [20], ELFE (Étude Longitudinale Française depuis l’Enfance, France, *n *= 9100 eligible children) [21], INMA (INfancia y Medio Ambiente Project, Spain, *n* = 1348 eligible children) [22] and SWS (Southampton Women’s Survey, United Kingdom, *n* = 134 eligible children) [23]. Further details on each cohort are provided in Online Resource 1.

### Preschool sleep duration

All cohorts measured child’s preschool sleep duration using different parental questionnaires (Online Resource 2 Table 1). Parents reported the time their child usually went to sleep (ALSPAC, ELFE, SWS) or to bed (EDEN) and woke up in the morning, as well as the duration of daytime naps. In INMA the parents were asked to provide night and daytime sleep duration.

**Table 1:** Characteristics of the participating study population

	ALSPAC (UK) 1991–1992		EDEN (France) 2003–2006		ELFE (France) 2011	INMA (Spain) 1997–2008	SWS (UK) 1998–2002
	Internalizing/Externalizing behavior	Language/ Non-verbal intelligence	Internalizing/Externalizing behavior	Language/ Non-verbal intelligence	Internalizing/Externalizing behavior	Language/Non-verbal intelligence	Language/ Non-verbal intelligence
*n*	3010/3009	718/719	876/877	865/866	8034	1285	111
% of original sample	20.0	4.8	46.1	45.5	44.3	60.2	3.5
**Child characteristics**							
Sex, male, *n* (%)	1517 (50.4)	383 (53.3)	467 (53.2)	469 (54.2)	4172 (51.9)	649 (50.5)	63 (56.8)
Birth weight, gr, mean (SD)	3435 (523)	3479 (512)	3309 (490)	3304 (494)	3353 (470)	3262 (452)	3461 (560)
Gestational age, weeks, mean (SD)	39.9 (1.7)	40.0 (1.5)	39.7 (1.6)	39.7 (1.6)	39.7 (1.4)	39.9 (1.4)	39.6 (2.0)
First born, yes, *n* (%)	1282 (42.6)	335 (46.6)	418 (47.7)	410 (47.3)	3684 (45.9)	739 (57.5)	63 (56.8)
Sleep duration, hours:min, mean (SD)	11:30 (0:54)	11:30 (0:53)	12:36 (0:57)	12:36 (0:57)	12:18 (0:44)	10:24 (0:57)	11:30 (0:51)
Age sleep duration measurement, years, mean (SD)	3.5 (0.1)	3.5 (0.1)	3.2 (0.1)	3.2 (0.1)	3.5 (0.2)	4.4 (0.2)	3.1 (0.1)
**Maternal characteristics**							
Maternal age at birth, years, mean (SD)	29.1 (4.5)	29.5 (4.2)	30.3 (4.4)	30.3 (4.5)	31.1 (4.5)	32.0 (4.0)	29.4 (3.4)
Mother born abroad, yes, *n* (%)	127 (4.2)	31 (4.3)	19 (2.2)	20 (2.3)	591 (7.4)	86 (6.7)	7 (6.3)
High maternal education level, *n* (%)	457 (15.2)	116 (16.2)	552 (62.9)	519 (60.0)	5792 (72.1)	473 (36.8)	34 (30.6)
Smoking in pregnancy, yes, *n* (%)	551 (19.6)	133 (18.5)	186 (21.3)	198 (22.9)	1231 (15.4)	383 (29.8)	17 (15.3)
Postpartum depression, yes, *n* (%)	236 (7.8)	48 (6.8)	67 (7.7)	67 (8.1)	658 (8.2)	NA	NA
**Household characteristics**							
EUSILC-based household income^a^, mean (SD)	7.1 (0.2)	7.1 (0.2)	7.4 (0.3)	7.4 (0.3)	7.5 (0.3)	7.1 (0.3)	7.3 (0.3)
Passive smoke exposure in the first year of life, yes, n (%)	986 (32.8)	205 (28.8)	364 (41.5)	364 (42.5)	2837 (35.3)	NA	17 (15.3)
**Outcome characteristics**							
Age at outcome measurement, years, mean (SD)	4.1 (0.1)	4.1 (0.03)	5.6 (0.1)	5.6 (0.1)	5.5 (0.5)	4.9 (0.6)	4.4 (0.1)
Outcome raw score^b^, mean (SD)	2.8 (2.3)/ 5.8 (3.2)^c^	101.1 (13.5) / 109.3 (14.5)	3.3 (2.5)/ 5.3 (3.7)	106.9 (13.7)/ 99.9 (13.5)	3.2 (2.6)/ 5.1 (3.3)	61.0 (15.6) / 53.7 (13.3)	111.3 (15.5)/ 105.2 (14.0)
Outcome percentile score^d^, mean (SD)	42.5 (30.3)/ 45.1 (29.3)	NA	50.0 (28.1) / 49.9 (28.6)	NA	42.8 (30.2) / 44.4 (29.3)	NA	NA
Outcome standardized score^d^, mean (SD)	NA	101.0 (14.8)/ 101.4 (14.9)	NA	100.0 (14.5)/ 100.6 (14.7)	NA	100.0 (14.9)/ 100.3 (14.6)	99.0 (15.4)/ 99.6 (15.5)

Data are given as mean (standard deviation) or number (percentage). Sample sizes are based on children with data on sleep duration, the specific outcome and all covariates

^a^Log-equivalised total disposable household income predicted using EUSILC data

^b^Behavior measured with the SDQ in all cohorts; language and non-verbal intelligence assessed by the WPPSI in ALSPAC, EDEN and SWS; assessed by the MSCA in INMA; the respective outcome names are displayed in the column header

^c^In ALSPAC internalizing raw score and externalizing raw score are available for 2944 and 2948 children, respectively

^d^The respective outcome names are displayed in the column header

*ALSPAC *Aavon Longitudinal Study of Parents and Children, *EDEN* Étude des Déterminants pré et postnatals du développement et de la santé de l’Enfant, *ELFE* Étude Longitudinale Française depuis l’Enfance, *INMA* INfancia y Medio Ambiente Project, *NA*  not available or not harmonized by the specific cohort, *SWS* Southampton Women’s Survey, *UK* United Kingdom

Cohorts harmonized total sleep duration in hours per day in preschool age (2-4 years) by summing nighttime and daytime sleep durations following a harmonization protocol. Sleep was assessed at a mean age of 3.1 years (SD: 0.1) in SWS, 3.2 years (SD: 0.1) in EDEN, 3.5 years (SD: 0.1, SD: 0.2) in ALSPAC and ELFE, respectively, and 4.4 years (SD: 0.2) in INMA.

To investigate a potential non-linear association between sleep duration and behavioral or cognitive outcomes, we categorized total sleep duration into thirds within each cohort based on tertiles (1st third includes children with the shortest sleep durations).

### Internalizing and externalizing behavior problems

Data on behavior was available in three cohorts: ALSPAC, EDEN and ELFE. All cohorts used the parent version of the Strength and Difficulties Questionnaire (SDQ) to measure internalizing and externalizing problems in children. The SDQ is a standardized questionnaire for children from 4 to 16 years with 25 items assessed on a three-point Likert scale [24]. The questionnaire covers five scales: emotional symptoms, peer problems, conduct problems, hyperactivity and prosocial behavior, ranging from 0 to 10 each [24]. The emotional and peer problems subscales were combined into the internalizing score, while the externalizing score includes the scales conduct and hyperactivity problems, as suggested for analyses in low-risk samples in the general population [25]. The SDQ is at least as good in detecting internalizing and externalizing problems compared to semi-structured interviews [26].

We used internalizing and externalizing percentile scores, which range from 0 to 100 and indicate the relative position of each child within his/her cohort and age group [17]. Higher percentile scores indicate more behavioral problems. Behavior was assessed at a mean age of 4.1 years (SD: 0.1) in ALSPAC, 5.5 years (SD: 0.5) in ELFE and 5.6 years (SD: 0.1) in EDEN.

### Language and non-verbal intelligence

Data on language and non-verbal intelligence were available in four cohorts: ALSPAC, EDEN, INMA and SWS. In ALSPAC, EDEN and SWS, language and non-verbal intelligence were assessed by trained psychologists using the verbal and performance intelligence scale of the Wechsler Preschool and Primary Scale of Intelligence (WPPSI). The WPPSI is an intelligence test for children aged 2 to 7 years that provides subtests on verbal and performance intelligence domains [27]. The verbal score includes the subtests Information, Vocabulary and Word Reasoning, while the performance score includes the subtests Block Design, Matrix Reasoning and Picture Concepts. In INMA, language and non-verbal intelligence were assessed by a psychologist using the verbal and perceptual-performance domains of the McCarthy Scales of Children’s Abilities (MSCA) [28]. This instrument is similar to the WPPSI and measures intelligence in children aged 2 to 8 years. The verbal scale consists of the subtests Pictorial Memory, Word Knowledge, Verbal Memory, Verbal Fluency and Opposite Analogies, while the perceptual-performance scale consists of the subtests Block Building, Puzzle Solving, Tapping Sequence, Right-left Orientation, Draw-a-design, Draw-a-child and Conceptual Grouping.

To allow comparison between the two tests, cohort-specific z-scores were calculated and standardized within each cohort to a mean of 100 and a SD of 15, following a harmonization protocol and the lead of other studies [29, 30]. Scores were measured at a mean age of 5.6 years (SD: 0.1) in EDEN and 4.9 years (SD: 0.6) in INMA. In ALSPAC (4.1 years (SD: 0.03)) and SWS (4.4 years (SD: 0.1)) they were measured in a subgroup of children.

### Covariates

Potential confounders were identified based on the literature and selected with creating directed acyclic graphs [31–34] (Online Resource 2 Fig. 1). 

The selected variables included sex, birthweight (kg), gestational age (weeks), birth order (first/later born), maternal age at birth (years), maternal education level according to International Standard Classification of Education 97/2011 (low/middle/high) [35, 36], whether the mother was born abroad (yes/no), maternal smoking in pregnancy (yes/no), the predicted equivalized total disposable household income at baseline [37], maternal postpartum depression (yes/no) (not harmonized in INMA/SWS) and child’s passive smoke exposure in the first year of life (yes/no) (not harmonized in INMA). Cohort-specific information on variable collection and missing data is shown in Online Resource 2 Tables 2–3.

### Statistical analyses

Analyses were performed in R (version 3.5.2) using DataSHIELD (version 6.1.0), a data analysis platform that enables federated analysis of data from different cohorts without physically sharing individual-level data [38–40].

**Fig. 1:**
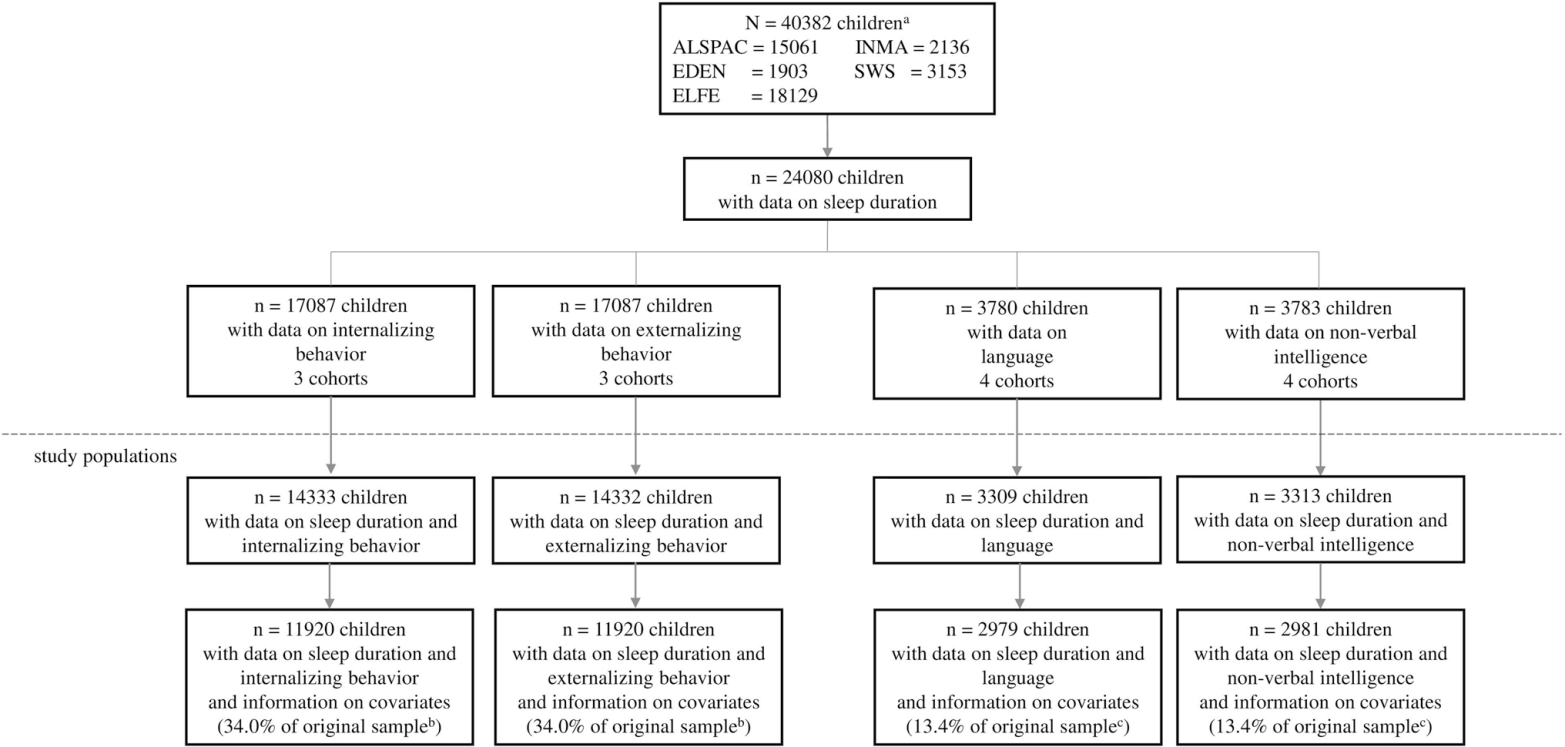
Flow chart illustrating participants included in the study ^a^N is based on all children with data on sex; ^b^The original sample for behavior analyses consists of data from ALSPAC, EDEN and ELFE: *N *= 35,093; ^c^The original sample for cognition analyses consists of data from ALSPAC, EDEN, INMA and SWS: *N* = 22,253. The same populations were used in both basic and adjusted models. *ALSPAC *Avon Longitudinal Study of Parents and Children, *EDEN* Étude des Déterminants pré et postnatals du développement et de la santé de l’Enfant, *ELFE* Étude Longitudinale Française depuis l’Enfance, *INMA* Infancia y Medio Ambiente Project, *SWS* Southampton Women’s Survey

We performed complete case analysis, including only participants with data on sleep, the specific outcome, and all covariates (Fig. 1). Of the 35,093 eligible children, 34.0% (11,920) had complete data for behavioral analyses, ranging from 20.0% in ALSPAC to 46.1% in EDEN. Of the 22,253 eligible children, 13.4% (2979–2981) had complete data for cognitive analyses, ranging from 3.5% in SWS to 60.2% in INMA.

We used two-stage individual participant data (IPD) meta-analysis to study the associations of sleep duration at age 3.5 years with behavioral and cognitive outcomes in children aged 5 years. Sleep duration was analyzed as continuous (decimal hours) and categorical variable (reference: 2nd third) to investigate the possibility that both shorter and longer sleep duration might be associated with the outcomes. For each outcome we constructed two models: a basic model adjusted for sex and age at outcome measurement and a model adjusted for other potential confounders. We conducted generalized linear regression analyses in each cohort and combined the effect estimates using random-effects meta-analysis. For this we used the “rma” command with the restricted maximum likelihood estimator of the “metafor” package in R. Heterogeneity between cohorts was described using *I*^2^ and *τ*^2^ [41].

We performed several sensitivity analyses: (1) using a one-stage IPD meta-analysis approach, (2) using raw scores of internalizing/externalizing behavior, (3) excluding twins and children with congenital malformation or cerebral palsy as this could possibly effect sleep, behavior and cognition, (4) adjusting for TV watching duration at preschool age, and (5) excluding INMA because of their later sleep measurement.

## Results

Table 1 shows the characteristics of the study population in each cohort divided by outcome. In both French cohorts mothers had higher education levels compared to mothers in the other cohorts. Children’s sleep duration differed between countries, with children from France showing a longer sleep duration than children from the UK or Spain. It should be noted, however, that children in INMA were older than children in the other cohorts. Overall mean sleep duration was 11h54min per day (SD: 1h01min) (Online Resource 2 Table 3).

Characteristics of the analyzed and excluded samples were different. Children in the analyzed sample had longer sleep durations, slightly lower behavior percentile scores and higher language or non-verbal intelligence scores than excluded children. Mothers in the analyzed sample had higher education levels, smoked less during pregnancy and were less likely to be born abroad compared to excluded mothers (Online Resource 2 Table 3).

### Associations between sleep duration and internalizing and externalizing behavior

**Fig. 2:**
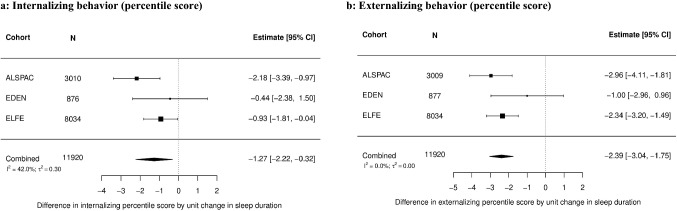
Association between total sleep duration per day at mean age of 3.5 years and 2a) internalizing behavior (percentile score), 2b) externalizing behavior (percentile score) at mean age of 5.1 years using two-stage IPD meta-analysis – adjusted models Adjusted for sex of the child, age at outcome measurement, maternal age at birth, maternal education, postpartum depression, mother born abroad, birthweight, gestational age, siblings position, passive smoke exposure in the first year of life, EUSILC-based household income, *ALSPAC* Avon Longitudinal Study of Parents and Children, *CI* Confidence interval, *EDEN* Étude des Déterminants pré et postnatals du développement et de la santé de l'Enfant, *ELFE* Étude Longitudinale Française depuis l'Enfance, *N* Number of children included in the analysis; *I*^2^ and *τ*^2^ statistics represent between cohort heterogeneity

**Fig. 3:**
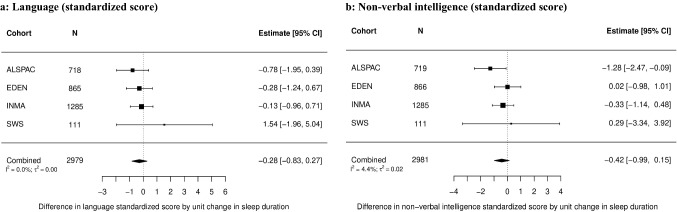
Association between total sleep duration per day at mean age of 3.7 years and 3a) language (standardized score), 3b) non-verbal intelligence (standardized score) at mean age of 4.9 years using two-stage IPD meta-analysis–adjusted model Adjusted for sex of the child, age at outcome measurement, maternal age at birth, maternal education, mother born abroad, birthweight, gestational age, siblings position, smoking in pregnancy, EUSILC-based household income ALSPAC: Avon Longitudinal Study of Parents and Children, *CI* confidence interval, *EDEN*: Étude des Déterminants pré et postnatals du développement et de la santé de l’Enfant, *INMA* INfancia y Medio Ambiente Project, *SWS* Southampton Women’s Survey, *N* number of children included in the analysis, *I*^2^ and *τ*^2^ statistics represent between cohort heterogeneity

Analyses examining the association between sleep duration and behavioral outcomes included 11,920 children from three cohorts (Fig. 1). Figs. 2a, b show that one hour of longer mean sleep duration per day at age 3.5 years was associated with lower internalizing and externalizing behavior percentile scores at 5.1 years (internalizing behavior: mean difference = − 1.27, 95% CI − 2.22, − 0.32; externalizing behavior: mean difference = – 2.39, 95% CI − 3.04, − 1.75). Heterogeneity between cohorts was moderate for internalizing behavior (*I*^2^ = 42.0%) and low for externalizing behavior (*I*^2^ = 0.0%) in adjusted models. ALSPAC showed a stronger negative association between sleep duration and behavioral outcomes than EDEN or ELFE. There was no evidence for a non-linear association between sleep duration and behavior (Online 2 Resource Table 9). Sensitivity analyses showed similar results (Online Resource 2 Tables 4–5, 8; Figs. 2–8).

### Sleep duration and language and non-verbal intelligence

Analyses investigating the association between sleep duration and language or non-verbal intelligence included 2979 and 2981 children, respectively, from four cohorts (Fig. 1). Figures 3a, b show trends of inverse associations between sleep duration at age 3.7 years with either language or non-verbal intelligence scores at 4.9 years, however, estimates were imprecise due to the relative small sample size and confidence intervals included null (language: mean difference = − 0.28, 95% CI − 0.83, 0.27; non-verbal intelligence: mean difference = − 0.42, 95% CI − 0.99, 0.15). Trends were mainly driven by ALSPAC, the oldest cohort. Between cohort heterogeneity in adjusted models was low (language: *I*^2^ = 0.0%, non-verbal intelligence: *I*^2^ = 4.4%). There was no evidence for a non-linear association between sleep duration and cognitive outcomes (Online Resource 2 Table 9). Sensitivity analyses delivered similar results (Online Resource 2 Tables 6–8; Figs. 9–13).

## Discussion

In this meta-analysis of IPD from five European cohorts, we observed that a longer mean sleep duration per day in preschool age was associated with lower subsequent scores of internalizing and externalizing behavior at 5 years of age, while the associations between sleep duration and language or non-verbal intelligence were imprecise with trends toward an inverse association.

Our results extend the knowledge from the few available longitudinal studies on the association of sleep duration with behavior in normally developing preschoolers [10–12]. In a Norwegian cohort (*N* = 32,662) a dose–response association was found between parent-reported short sleep duration (≤ 10 h, 11-12 h vs. ≥ 13 h) at 18 months and the risk of internalizing and externalizing problems at age 5 years assessed by the Child Behavior Checklist [10]. Jansen et al. [11] showed that parent-reported sleep duration of less than 12.5 h at age 2 years was a risk factor for anxiety or depressive symptoms at age 3 years measured with the Child Behavior Checklist in 4782 children. In a sample of 1492 children a short sleep duration pattern before the age of 3.4 years was associated with higher hyperactivity-impulsivity scores at age 6 years [12]. All mentioned studies adjusted for pre-existing behavioral symptoms, to account at least partially for reverse causality, because pre-existing behavioral and cognitive traits are likely to influence sleep duration and correlate with equivalent traits at older ages [42]. Outcome at time of exposure measurement and exposure at time of outcome measurement were not available in the present study. Outcome misclassification needs to be additionally considered, as parents of children with more behavioral problems at an earlier age might report sleep duration as shorter than it is. This should be taken into account when interpreting our results.

The effect estimates obtained for internalizing and externalizing behavior percentile scores in our study were relatively small. Even though this difference may not be clinically relevant, it may reflect large differences at the population-level. Experimental studies with young children showed that even light levels of sleep deprivation over just a few days can impair the ability of emotion- and self-regulation, which are potential risk factors for problem behavior [43, 44].

There are some biological mechanisms that may explain the associations of sleep and behavioral outcomes. A systematic review of sleep and its associations with brain functions and structures in children suggested for example that shorter sleep duration is associated with greater reactivity in brain regions that are involved in emotion processing [45]. Also studies in adults showed that sleep deprivation led to a stronger amygdala response to negative and neutral emotional images [46, 47]. This could result in less cognitive control over emotion processing leading to more irritability and negative affect [48]. In our study, we found an association with internalizing and externalizing problems which are closely related to emotional processes.

Previous studies reported mixed results of the association between sleep duration and cognition in preschool children [12–14, 49]. Touchette et al. [12] reported that children with persistently short sleep durations during preschool age scored lower on the Peabody Picture Vocabulary test at age 5 years, and children with a short sleep duration pattern before the age of 3.4 years had lower non-verbal intelligence skills assessed with the Wechsler Intelligence Scale at age 6 years. In contrast to our study, where only one time-point was analyzed, Touchette et al. [12] measured sleep at five time-points and created sleep patterns. Another study in 2800 children reported that children sleeping within the recommended sleep duration range of 11 to 14 h at age 2 years had better non-verbal intelligence as well as language scores at age 6 years than children with shorter or longer sleep [14]. Authors concluded that children with average sleep duration also most likely have normal levels in other developmental areas such as cognitive outcomes. Dionne et al. [13] showed in a sample of 1029 children that parental reports of night sleep duration at 30 months were not associated with receptive vocabulary assessed by the Peabody Picture Vocabulary Test at age 5 years, but with a higher day/night sleep ratio at 18 months, indicating less mature sleep consolidation. A study in 194 children showed a trend of an inverse association of mother-reported sleep duration at 24 months with verbal and non-verbal intelligence at age 3 years measured with the WPPSI [49].

The different findings show that further longitudinal studies with multiple sleep duration measurements, other sleep variables as day/night sleep ratio and larger sample sizes are needed to get a clearer picture of this potential relationship.

### Strengths and limitations

Our study’s major strength is the federated analysis approach which allowed analyses of IPD from five cohorts including children from three European countries. The consistent harmonization of variables between cohorts as well as the consistent adjustment for confounders in the analyses reduced between-study heterogeneity and strengthens reproducibility of the findings across cohorts. Another strength is that outcomes were measured with validated questionnaires (SDQ) and tests performed by trained psychologists (WPPSI, MCSA).

One limitation of our study is the complete case analysis. For behavioral analyses 34.0% of the original sample contributed, whereas this was just 13.4% for cognitive outcomes, in part because language and non-verbal intelligence were measured only in subgroups in ALSPAC and SWS. This potential loss of information leads to loss of statistical power and increases the uncertainty of the estimates. Complete case analysis assumes that the chance of being a complete case is independent of the outcome after adjusting for covariates [50]. We acknowledge that with the amount of missing data and the demonstrated differences between those included and not, it is plausible that selection bias has had some influence on our findings.

Sleep duration was based on parental reports in all cohorts. Studies have shown the tendency of parents to overestimate their child’s real sleep duration compared to device-based measured sleep [51, 52]. While questions used to measure sleep duration were different across cohorts, the mean sleep duration in our study was similar to values in a meta-analysis of preschoolers (mean 11h54min) [53] and is within the range of 10 to 13 h recommended by the American Academy of Sleep Medicine for children aged 3 to 5 years [3], suggesting that it is rather cultural background that might play an important role in the specific country differences. The variation in sleep duration between the three countries that contributed to this study, are consistent with other studies showing that children from northern and middle European countries sleep longer than children in southern or eastern Europe [54, 55].

Methodological aspects in data acquisition might have affected the measured sleep duration, outcomes and covariates. However, great efforts were undertaken to harmonize data between cohorts [15–17]. The variable catalog with data source information is openly available at https://data-catalogue.molgeniscloud.org/catalogue/catalogue/#/networks-catalogue/EUChildNetwork/variables. The downside of the federated analysis approach is that it tends to use the lowest common denominator of available information for data harmonization, which can lead to residual confounding. Many confounders were reduced to binary variables (for example passive smoking (yes/no), birth order (first/later born) etc.) and ethnicity was approximated by whether the mother was born abroad or not, which will capture only a modest part of the complex influence of confounders on child sleep and outcomes.

## Conclusion

Using IPD from five European cohorts, we showed that longer sleep duration at 3.5 years of age was associated with both lower internalizing and externalizing problem behavior scores at 5 years of age, while the evidence of an association of sleep duration with either language or non-verbal intelligence was imprecise. Our results suggest that longer sleep duration at early preschool ages may be important for later behavioral outcomes. These findings could be due to confounding or reverse causality and need replication.

### Supplementary Information

Below is the link to the electronic supplementary material.

Supplementary file1 (PDF 415 KB)

Supplementary file2 (PDF 1400 KB)

## Data Availability

The data used in the analysis is not freely available as access is managed by each individual cohort. Researchers who want to use data from the EU Child Cohort Network can send a request to lifecycle@erasmusmc.nl (more information under https://lifecycle-project.eu/).

## Abbreviations

ALSPAC: Avon Longitudinal Study of Parents and Children
CI: Confidence interval
EDEN: Étude des Déterminants pré et postnatals du développement et de la santé de l’Enfant
ELFE: Étude Longitudinale Française depuis l’Enfance
IPD: Individual participant data
INMA: INfancia y Medio Ambiente Project
MSCA: McCarthy Scales of Children’s Abilities
SD: Standard deviation
SDQ: Strengths and Difficulties Questionnaire
SWS: Southampton Women’s Survey
WPPSI: Wechsler Preschool and Primary Scale of Intelligence

## Code availability

Statistical analysis was performed in R (version 3.5.2) using DataSHIELD (version 6.1.0).
