# Supplementary material for: Sleep duration in preschool age and later behavioral and cognitive outcomes: an individual participant data meta-analysis in five European cohorts
Source: Eur Child Adolesc Psychiatry. 2023 Feb 7;33(1):167–77. doi: 10.1007/s00787-023-02149-0 (PMC10805899; doi:10.1007/s00787-023-02149-0)
Supplement: Supplementary file 1 — Supplementary file1 (PDF 415 KB) [file 787_2023_2149_MOESM1_ESM.pdf]

Supplementary Information 1

Cohort's description, ethical approval, acknowledgements and funding

Article title: Sleep duration in preschool age and later behavioral and cognitive outcomes: an individual participant data meta-analysis in five European cohorts

Journal name: European Child & Adolescent Psychiatry

Author names: Kathrin Guerlich, Demetris Avraam, Tim Cadman, Lucinda Calas, Marie-Aline Charles, Ahmed Elhakeem, Silvia Fernández-Barrés, Mònica Guxens, Barbara Heude, Jesús Ibarluzea, Hazel Inskip, Jordi Julvez, Deborah A Lawlor, Mario Murcia, Theodosia Salika, Jordi Sunyer, Muriel Tafflet, Berthold Koletzko, Veit Grote*, Sabine Plancoulaine*
*equal contribution

Address correspondence to:

Veit Grote, Division of Metabolic and Nutritional Medicine, Dr. von Hauner Children's Hospital, LMU University Hospital, Lindwurmstr. 4, D-80337 München. Email: veit.grote@med.uni-muenchen.de.

Sabine Plancoulaine, CRESS U1153, èquipe EARoH – 16 Avenue Paul Vaillant-Couturier, F-94807 Villejuif cedex. Email: sabine.plancoulaine@inserm.fr.

Cohort's description, ethical approval, acknowledgements and funding

Avon Longitudinal Study of Parents and Children (ALSPAC)

Cohort profile: Fraser A, Macdonald-Wallis C, Tilling K, Boyd A, Golding J, Davey Smith G, Henderson J, Macleod J, Molloy L, Ness A, Ring S, Nelson SM, Lawlor DA (2013) Cohort Profile: the Avon Longitudinal Study of Parents and Children: ALSPAC mothers cohort. Int J Epidemiol 42 (1):97-110. doi:10.1093/ije/dys066; Boyd A, Golding J, Macleod J, Lawlor DA, Fraser A, Henderson J, Molloy L, Ness A, Ring S, Davey Smith G (2013) Cohort Profile: the 'children of the 90s'--the index offspring of the Avon Longitudinal Study of Parents and Children. Int J Epidemiol 42 (1):111-127. doi:10.1093/ije/dys064

Design and study population: Pregnant women resident in Avon, UK with expected dates of delivery 1st April 1991 to 31st December 1992 were invited to take part in the study. The initial number of pregnancies enrolled is 14,541 (for these at least one questionnaire has been returned or a "Children in Focus" clinic had been attended by 19/07/99). Of these initial pregnancies, there was a total of 14,676 foetuses, resulting in 14,062 live births and 13,988 children who were alive at 1 year of age.
When the oldest children were approximately 7 years of age, an attempt was made to bolster the initial sample with eligible cases who had failed to join the study originally. As a result, when considering variables collected from the age of seven onwards (and potentially abstracted from obstetric notes) there are data available for more than the 14,541 pregnancies mentioned above. The number of new pregnancies not in the initial sample (known as Phase I enrolment) that are currently represented on the built files and reflecting enrolment status at the age of 24 is 913 (456, 262 and 195 recruited during Phases II, III and IV respectively), resulting in an additional 913 children being enrolled. The phases of enrolment are described in more detail in the cohort profile paper and its update. The total sample size for analyses using any data collected after the age of seven is therefore 15,454 pregnancies, resulting in 15,589 foetuses. Of these 14,901 were alive at 1 year of age.
A 10% sample of the ALSPAC cohort, known as the Children in Focus (CiF) group, attended clinics at the University of Bristol at various time intervals between 4 to 61 months of age. The CiF group were chosen at random from the last 6 months of ALSPAC births (1432 families attended at least one clinic). Excluded were those mothers who had moved out of the area or were lost to follow-up, and those partaking in another study of infant development in Avon.
Please note that the study website contains details of all the data that is available through a fully searchable data dictionary and variable search tool: http://www.bristol.ac.uk/alspac/researchers/our-data/

Consent and ethical approval: Ethical approval for the study was obtained from the ALSPAC Ethics and Law Committee and the Local Research Ethics Committees. Informed consent for the use of data collected via questionnaires and clinics was obtained from participants following the recommendations of the ALSPAC Ethics and Law Committee at the time.
More details on the ethics committee/institutional review board(s) can be found here: http://www.bristol.ac.uk/alspac/researchers/research-ethics/

Study funding: The UK Medical Research Council and Wellcome (Grant ref: 217065/Z/19/Z) and the University of Bristol provide core support for ALSPAC. This publication is the work of the authors and they will serve as guarantors for the contents of this paper. A comprehensive list of grants funding is available on the ALSPAC website (http://www.bristol.ac.uk/alspac/external/documents/grant-acknowledgements.pdf).
The funders had no role in the design of the study, the collection, analysis, or interpretation of

the data; the writing of the manuscript, or the decision to submit the manuscript for publication. The views expressed in this paper are those of the authors and not necessarily those of any funder.

Acknowledgements: We are extremely grateful to all of the families who took part in this study, the midwives for their help in recruiting them, and the whole ALSPAC team, which includes interviewers, computer and laboratory technicians, clerical workers, research scientists, volunteers, managers, receptionists and nurses.

Etude des Déterminants pré et post natals précoces du développement psychomoteur et de la santé de l'Enfant (EDEN)

Cohort profile: Heude B, Forhan A, Slama R, Douhaud L, Bedel S, Saurel-Cubizolles MJ, Hankard R, Thiebaugeorges O, De Agostini M, Annesi-Maesano I, Kaminski M, Charles MA, group Em-ccs (2016) Cohort Profile: The EDEN mother-child cohort on the prenatal and early postnatal determinants of child health and development. Int J Epidemiol 45 (2):353-363. doi:10.1093/ije/dyv151

Design and study population: The EDEN Mother-Child Cohort is a longitudinal cohort study and was set up in 2003 in two university maternity clinics, in Nancy and Poitiers, France. Pregnant women seen for a prenatal visit at the departments of Obstetrics and Gynecology of the University Hospital of Nancy and Poitiers before their twenty-fourth week of amenorrhea were invited to participate. Enrolment started in February 2003 in Poitiers and September 2003 in Nancy; it lasted 27 months in each centre. Among eligible women, 55% (n=2002) accepted to participate. The children's health and behavior was regularly followed-up for up to 8 years from birth, by visits to research centres and questionnaires mailed to parents. Exclusion criteria were multiple pregnancies, known diabetes before pregnancy, French illiteracy or planning to move out of the region within the next 3 years. More information on EDEN can be found at its website (https://eden.vjf.inserm.fr/index.php?lang=en).

Consent and ethical approval: The study received approval from the ethics committee (CCPPRB) of Kremlin Bicêtre on 12 December 2002 and from CNIL (Commission Nationale Informatique et Liberté), the French data privacy institution. All subjects gave their informed consent for inclusion before they participated in the study. Consent for the child was obtained from both parents after the child's birth.

Study funding: Foundation for Medical Research (FRM), National Agency for Research (ANR), National Institute for Research in Public Health (IRESP: TGIR cohorte santé 2008 program), French Ministry of Health (DGS), French Ministry of Research, Inserm Bone and Joint Diseases National Research (PRO-A) and Human Nutrition National Research Programs, Paris–Sud University, Nestlé, French National Institute for Population Health Surveillance (InVS), French National Institute for Health Education (INPES), the European Union FP7 programmes (FP7/2007-2013, HELIX, ESCAPE, ENRIECO, MEDall projects), Diabetes National Research Program (through a collaboration with the French Association of Diabetic Patients (AFD)), French Agency for Environmental Health Safety (now ANSES), MutuelleGénérale de l'EducationNationale (MGEN), French National Agency for Food Security, Health and Environment-wide Associations based on Large population Surveys (HEALS) and the French-speaking association for the study of diabetes and metabolism (ALFEDIAM). The funders had no role in study design, data collection and analysis, decision to publish, or preparation of the manuscript.

Acknowledgements: We are indebted to all the children and their parents for participation, as well as to the research nurses, research assistants, and laboratory personnel involved in the EDEN study. The EDEN Mother–Child Cohort Study Group includes: I. Annesi-Maesano, J.Y Bernard, J. Botton, M.A. Charles, P. Dargent-Molina, B. de Lauzon-Guillain, P. Ducimetière, M. de Agostini, B. Foliguet, A. Forhan, X. Fritel, A. Germa, V. Goua, R. Hankard, B. Heude, M. Kaminski, B. Larroque†, N. Lelong, J. Lepeule, G. Magnin, L. Marchand, C. Nabet, F. Pierre, R. Slama, M.J. Saurel-Cubizolles, M. Schweitzer, O. Thiebaugeorges.

Etude Longitudinale Francaise depuis l'Enfance (ELFE)

Cohort profile: Charles MA, Thierry X, Lanoe J-L, Bois C, Dufourg M-N, Popa R, Cheminat M, Zaros C, Geay B (2019) Cohort Profile: The French national cohort of children (ELFE): birth to 5 years. Int J Epidemiol 49 (2):368-369j. doi:10.1093/ije/dyz227

Design and study population: ELFE is a prospective multidisciplinary nationally representative birth-cohort study. It included 18,329 newborn infants in a random sample of 349 maternity units in 2011 with the aim of examining many aspects of the lives of these children from the perspectives of health, social sciences and environmental health up to adulthood. Inclusion took place during 25 selected recruitment days over four waves comprising 4 to 8 days each, from April 2011 and covering all four seasons. Inclusion criteria were infants born after 33 weeks' gestation to mothers ≥ 18 years older and who were not planning to move outside of Metropolitan France in the following 3 years and were able to read French, Arabic, Turkish, or English. More details can be found at its website (https://www.elfe-france.fr/en/).

Consent and ethical approval: Ethical approvals for data collection in maternity units and for each data collection wave during follow-up were obtained from the national advisory committee on information processing in health research (CCTIRS: Comité Consultatif sur le Traitement de l'Information en matière de Recherche dans le domaine de la Santé), the national data protection authority (CNIL: Comission Nationale Informatique et Liberté) and, in case of invasive data collection such as biological sampling, the committee for protection of persons engaged in research (CPP: Comité de Protection des Personnes). The ELFE study was also approved by the national committee for statistical information (CNIS: Conseil National de l'Information Statistique). Informed consent was signed by the parents or the mother alone, with the father being informed of his right to deny consent for participation.

Study funding : The Elfe cohort is a joint project between the Institut national des études démographiques (Ined) and the Institut national de la santé et de la recherche médicale (Inserm), in partnership with the Établissement français du sang (EFS), Santé publique France, the Institut national de la statistique et des études économiques (Insee), the Direction générale de la santé (DGS, part of the Ministry of Health and Social Affairs), the Direction générale de la prévention des risques (DGPR, Ministry for the Environment), the Direction de la recherche, des études, de l'évaluation et des statistiques (DREES, Ministry of Health and Social Affairs), the Département des études, de la prospective et des statistiques (DEPS, Ministry of Culture), and the Caisse nationale des allocations familiales (CNAF), with the support of the Ministry of Higher Education and Research and the Institut national de la jeunesse et de l'éducation populaire (INJEP). Via the RECONAI platform, it receives a government grant managed by the Agence nationale de la recherche under the "Investissements d'avenir" programmes (ANR-11-EQPX-0038 and ANR-19-COHO-001).

Acknowledgements : The Elfe survey is a joint production of the Institut national d'études démographiques (Ined), the Institut national de la santé et de la recherche médicale (Inserm), the Établissement français du sang (EFS), Santé publique France, the Institut national de la statistique et des études économiques (Insee), the Direction générale de la santé (DGS, Ministry of Health), the Direction générale de la prévention des risques (DGPR, Ministry of the Environment) the Department of Research, Studies, Evaluation and Statistics (Drees, Ministry of Health and Social Affairs), the Department of Studies, Forecasting and Statistics (DEPS, Ministry of Culture) and the National Family Allowances Fund (Cnaf), with the support of the Ministry of Higher Education, Research and Innovation and the National Institute for Youth and Popular Education (INJEP).

INfancia y Medio Ambiente Project (INMA)

Cohort profile: Guxens M, Ballester F, Espada M, Fernandez MF, Grimalt JO, Ibarluzea J, Olea N, Rebagliato M, Tardon A, Torrent M, Vioque J, Vrijheid M, Sunyer J, Project I (2012) Cohort Profile: the INMA--INfancia y Medio Ambiente (Environment and Childhood) Project. Int J Epidemiol 41 (4):930-940. doi:10.1093/ije/dyr054

Design and study population: The INMA–INfancia y Medio Ambiente (Environment and Childhood) Project is a network of birth cohorts in Spain. The INMA project is a prospective population-based cohort study investigating the associations between pre- and post-natal environmental exposures and growth, health and development from early fetal life until adolescence. Women during weeks 6–10 of pregnancy with a singleton living in seven different Spanish regions Ribera d'Ebre, Menorca, Granada, Valencia, Sabadell, Asturias and Gipuzkoa) were eligible for inclusion from March 1997 to January 2008. Women who resided or intended to deliver outside the study area, who were aged under 16 years, who had twin or multiple pregnancies, who had assisted reproduction or who had communication problems were excluded. More information can be found at its website (http://www.proyectoinma.org/)

Consent and ethical approval : Written informed consent was obtained from all participating parents. The study was approved by the Ethical Committee of the Municipal Institute of Medical Investigation and by the Ethical Committee of the hospitals involved in the study.

Study funding: INMA-Valencia was funded by Grants from UE (FP7-ENV-2011 cod 282957 and HEALTH.2010.2.4.5-1), Spain: ISCIII (G03/176; FIS-FEDER: PI09/02647, PI11/01007, PI11/02591, PI11/02038, PI13/1944, PI13/2032, PI14/00891, PI14/01687, and PI16/1288; Miguel Servet-FEDER CP11/00178, CP15/00025, and CPII16/00051), and Generalitat Valenciana: FISABIO (UGP 15-230, UGP-15-244,and UGP-15-249). INMA-Gipuzkoa was funded by grants from the Instituto de Salud Carlos III (FISFIS PI06/0867, FISPS09/0009) 0867,Red INMA G03/176) and the Departamento de Salud del Gobierno Vasco (2005111093 and 2009111069) and the Provincial Government of Guipúzcoa (DFG06/004 and FG08/001). INMA-Sabadell was funded by grants from ISCIII (Red INMA G03/176; CB06/02/0041; FIS-FEDER: PI041436; PI081151; PI12/01890; CP13/00054; PI15/00118; CP16/00128; PI16/00118; PI16/00261; PI18/00547), CIBERESP, Generalitat de Catalunya-CIRIT (1999SGR 00241), Generalitat de Catalunya-AGAUR (2009 SGR 501, 2014 SGR 822), Fundació La Marató de TV3 (090430), Spanish Ministry of Economy and Competitiveness (SAF2012-32991 incl. FEDER funds), Agence Nationale de Securite Sanitaire de l'Alimentation de l'Environnement et du Travail (1262C0010; EST-2016 RF-21), and the European Commission (261357, 308333, 603794 and 634453). ISGlobal acknowledges support

from the Spanih Ministry of Science, Innovation and Universities through the "Centro de Excelencia Severo Ochoa 2019-2023" Program (CEX2018-000806-S), and support from the Generalitat de Catalunya through the CERCA Program.

Acknowledgements: The authors would particularly like to thank all the participants for their generous collaboration. The authors are grateful to Silvia Fochs, Nuria Pey, Mireia Garcia, Maria Victoria Estraña, Maria Victoria Iturriaga, Cristina Capo and Josep LLuch for their assistance in contacting the families and administering the questionnaires.

Southampton Women's Survey (SWS)

Cohort profile: Inskip HM, Godfrey KM, Robinson SM, Law CM, Barker DJ, Cooper C, Group SWSS (2006) Cohort profile: The Southampton Women's Survey. Int J Epidemiol 35 (1):42-48. doi:10.1093/ije/dyi202

Design and study population: SWS is a population-based prospective birth cohort study of 12 583, initially non-pregnant, women aged 20–34 years, living in Southampton, UK. Assessments of lifestyle, diet and anthropometry were performed at study entry in 1998–2002. Women who subsequently became pregnant, were followed up during pregnancy; and their offspring have been studied in infancy and childhood.

Consent and ethical approval: SWS study was conducted according to the guidelines laid down in the Declaration of Helsinki and was approved by the Southampton and Southwest Hampshire Local Research Ethics Committee (06/Q1702/104). Written informed consent was obtained from all participating women and by a parent or guardian with parental responsibility on behalf of their children.

Study funding: The SWS is supported by grants from the Medical Research Council, National Institute for Health Research Southampton Biomedical Research Centre, British Heart Foundation, University of Southampton and University Hospital Southampton National Health Service Foundation Trust, and the European Union's Seventh Framework Programme (FP7/2007-2013), project EarlyNutrition (grant 289346). Study participants were drawn from a cohort study funded by the Medical Research Council and the Dunhill Medical Trust. HMI's salary is paid by the UK Medical Research Council.

Acknowledgements: The authors are grateful to the women of Southampton who gave their time to take part in the Southampton Women's Survey and to the research nurses and other staff who collected and processed the data.
