# Supplementary material for: Sleep duration in preschool age and later behavioral and cognitive outcomes: an individual participant data meta-analysis in five European cohorts
Source: Eur Child Adolesc Psychiatry. 2023 Feb 7;33(1):167–77. doi: 10.1007/s00787-023-02149-0 (PMC10805899; doi:10.1007/s00787-023-02149-0)
Supplement: Supplementary file 2 — Supplementary file2 (PDF 1400 KB) [file 787_2023_2149_MOESM2_ESM.pdf]

Supplementary Information 2:

Tables and Figures

Article title: Sleep duration in preschool age and later behavioral and cognitive outcomes: an individual participant data meta-analysis in five European cohorts

Journal name: European Child & Adolescent Psychiatry

Author names: Kathrin Guerlich, Demetris Avraam, Tim Cadman, Lucinda Calas, Marie-Aline Charles, Ahmed Elhakeem, Silvia Fernández-Barrés, Mònica Guxens, Barbara Heude, Jesús Ibarluzea, Hazel Inskip, Jordi Julvez, Deborah A Lawlor, Mario Murcia, Theodosia Salika, Jordi Sunyer, Muriel Tafflet, Berthold Koletzko, Veit Grote*, Sabine Plancoulaine*
*equal contribution

Address correspondence to:

Veit Grote, Division of Metabolic and Nutritional Medicine, Dr. von Hauner Children's Hospital, LMU University Hospital, Lindwurmstr. 4, D-80337 München. Email: veit.grote@med.uni-muenchen.de.

Sabine Plancoulaine, CRESS U1153, èquipe EARoH – 16 Avenue Paul Vaillant-Couturier, F-94807 Villejuif cedex. Email: sabine.plancoulaine@inserm.fr.

Table of Contents

Tables **4**

Table 1: Details of sleep duration measurement in participating cohorts and harmonization procedures 4

Table 2: Details of data collected on covariates in participating cohorts 6

Table 3: Characteristics of the participating cohort population: Missing data and comparison of analyzed sample vs. excluded sample 7

Table 4: Association between total sleep duration per day at mean age of 3.5 years and internalizing/externalizing behavior (percentile and raw score) at mean age of 5.1 years using one-stage IPD meta-analysis – basic models 9

Table 5: Association between total sleep duration per day at mean age of 3.5 years and internalizing/externalizing behavior (percentile and raw score) at mean age of 5.1 years using one-stage IPD meta-analysis – adjusted models 9

Table 6: Association between total sleep duration per day at mean age of 3.7 years and language/non-verbal intelligence (standardized score) at mean age of 4.9 years using one-stage IPD meta-analysis – basic models 10

Table 7: Association between total sleep duration per day at mean age of 3.7 years and language/non-verbal intelligence (standardized score) at mean age of 4.9 years using one-stage IPD meta-analysis – adjusted models 10

Table 8: Associations between sleep tertiles and internalizing behavior, externalizing behavior, language and non-verbal intelligence using two-stage IPD meta-analysis – basic models 11

Table 9: Associations between sleep tertiles and internalizing behavior, externalizing behavior, language and non-verbal intelligence using two-stage IPD meta-analysis – adjusted models 11

Figures **12**

Figure 1: Directed acyclic graphs illustrating the variables required to control for confounding between sleep duration and 1a) problem behavior, 1b) cognitive outcomes 12

Figure 2: Association between total sleep duration per day at mean age of 3.5 years and 2a) internalizing behavior (raw score), 2b) externalizing behavior (raw score) at mean age of 5.1 years using two-stage IPD meta-analysis – basic models 14

Figure 3: Association between total sleep duration per day at mean age of 3.5 years and 3a) internalizing behavior (percentile score), 3b) externalizing behavior (percentile score) at mean age of 5.1 years using two-stage IPD meta-analysis – basic models 15

Figure 4: Association between total sleep duration per day at mean age of 3.5 years and internalizing behavior (raw score) at mean age of 5.1 years using two-stage IPD meta-analysis – adjusted models ... 16

Figure 5: Association between total sleep duration per day at mean age of 3.5 years and internalizing behavior (percentile score) at mean age of 5.1 years using two-stage IPD meta-analysis – adjusted models 18

Figure 6: Association between total sleep duration per day at mean age of 3.5 years and externalizing behavior (raw score) at mean age of 5.1 years using two-stage IPD meta-analysis – adjusted models ... 19

Figure 7: Association between total sleep duration per day at mean age of 3.5 years and externalizing behavior (percentile score) at mean age of 5.1 years using two-stage IPD meta-analysis – adjusted models 21

Figure 8: Association between total sleep duration per day at mean age of 3.5 years and 8a) internalizing behavior (percentile score), 8b) externalizing behavior (percentile score) at mean age of 5.1 years using two-stage IPD meta-analysis: Twins and children with congenital malformation, cerebral palsy excluded from analysis – adjusted models 22

Figure 9: Association between total sleep duration per day at mean age of 3.7 years and 9a) language (standardized score), 9b) non-verbal intelligence (standardized score) at mean age of 4.9 years using two-stage IPD meta-analysis – basic models 23

Figure 10: Association between total sleep duration per day at mean age of 3.7 years and language (standardized score) at mean age of 4.9 years using two-stage IPD meta-analysis – adjusted models ... 24

Figure 11: Association between total sleep duration per day at mean age of 3.7 years and non-verbal intelligence (standardized score) at mean age of 4.9 years using two-stage IPD meta-analysis – adjusted models 25

Figure 12: Association between total sleep duration per day at mean age of 3.7 years and 12a) language (standardized score), 12b) non-verbal intelligence (standardized score) at mean age of 4.9 years using two-stage IPD meta-analysis: Twins and children with congenital malformation excluded from analysis – adjusted models.. 26

Figure 13: Association between total sleep duration per day at mean age of 3.7 years and 13a) language (standardized score), 13b) non-verbal intelligence (standardized score) at mean age of 4.9 years using two-stage IPD meta-analysis: INMA excluded from analysis – adjusted models 27

Tables

Table 1: Details of sleep duration measurement in participating cohorts and harmonization procedures

All harmonization manuals can be downloaded from the LifeCycle website (https://lifecycle-project.eu).

Cohort (country)	Sleep duration
ALSPAC (UK)	Parental questionnaire at age 3.5 years<br><br>Nighttime sleep<br>“Normally what time in the evening does your child go to sleep?” (Unit is time expressed in 24 hour format)<br>“What time does he/she normally wake up in the morning?” (Unit is time expressed in 24 hour format)<br>➔ From bed time and wake time, sleep duration per night in hours was calculated<br><br>Daytime naps<br>“How many hours sleep does he/she usually have during the day time?”<br>Categorical: None, less than 1 hour, 1-2 hours, more than 2 hours, don’t know<br>➔ Categorical variables were transformed in a numerical variable, the middle of the interval was taken<br>None <- 0<br>Less than 1 hour <- 0.5<br>1-2 hours <- 1.5<br>More than 2 hours <- 2.5<br>Don’t know <- NA<br><br>Harmonization:<br>➔ Time spent sleeping in hours per usual day:<br>Total sleep time/night (hours) + nap time/day (hours)
EDEN (France)	Parental questionnaire at age 3 years<br><br>Nighttime sleep<br>“Usually, at what time does he/she go to bed?”<br>“Usually, at what time does he/she wake up?”<br>➔ From bed time and wake, time sleep duration per night (hours, mins) was calculated<br><br>Daytime naps<br>“For how long does he/she usually nap? (hours, mins)”<br>➔ Average daily nap time (hours, mins)<br><br>Harmonization:<br>➔ Time spent sleeping in hours per usual day:<br>Total sleep time/night (hours) + nap time/day (hours)
ELFE (France)	Parental questionnaire at age 3.5 years<br><br>Nighttime sleep<br>“At what time does he/she go to sleep at night, on usual weekdays?”<br>“At what time does he/she go to sleep at night, on usual weekend or vacation days?”<br>“At what time does he/she wake up in the morning, on usual weekdays?”<br>“At what time does he/she wake up in the morning, on usual weekend or vacation days?”<br>➔ From bed time and wake times, sleep duration per night (hours, mins) was calculated<br>Daytime naps<br>“Does he nap during the day on usual weekdays?”<br>“For how long does he/she usually nap on usual weekdays?”<br>“Does he/she nap during the day on usual weekend or vacation days?”

	"For how long does he/she usually nap on weekend or vacation days?"<br>➔ Average daily nap time (hours,mins)<br><br>Harmonization:<br>➔ Time spent sleeping in hours per day:<br>(((total sleep time/night on a weekday (hours) + nap time/day on a weekday (hours))*5) + ((total sleep time/night on a weekend-day (hours) + nap time/day on a weekend-day (hours))*2)) / 7
INMA (Spain)	Parental questionnaire at age 4 years<br><br>"How many hours does your child sleep during the week (h/day)?" (night and during naps)<br>"How many hours does your child sleep during the weekend (h/day)?" (night and during naps)<br><br>Harmonization:<br>➔ Time spent sleeping in hours per day:<br>➔ ((weekday sleep time (hours)*5)+(weekend sleep time (hours)*2)) / 7
SWS (UK)	Parental questionnaire at age 3 years<br><br>Nighttime sleep<br>"What time does the study child generally go to sleep at night? (24hr clock)"<br>__ __ __ __<br>"How many times per night does he/she generally wake for any reason? Please answer this in relation to the last month?"<br>__ . __ per night<br>"In total, how long is he/she generally awake? (Only record if regularly over 30mins)"<br>__ __ hrs __ __ mins per night<br>"What time does he/she generally wake up in the morning? (24hr clock)"<br>__ __ __ __<br>➔ From responses sleep duration per night in hours was calculated<br><br>Daytime naps<br>"How many days per week does he/she take a daytime nap? Please answer this in relation to the last month?"<br>__<br>"On the days he/she naps, what is the total time spent napping during the day?"<br>__ hrs __ __ mins<br>➔ Average daily nap time<br><br>Harmonization:<br>➔ Time spent sleeping in hours per usual day:<br>Total sleep time/night (hours) + average nap time/day (hours)

Abbreviations: ALSPAC: Avon Longitudinal Study of Parents and Children; EDEN: Étude des Déterminants pré et postnatals du développement et de la santé de l'Enfant; ELFE: Étude Longitudinale Française depuis l'Enfance; INMA: INfancia y Medio Ambiente Project; SWS: Southampton Women's Survey

Table 2: Details of data collected on covariates in participating cohorts

Covariates	ALSPAC	EDEN	ELFE	INMA	SWS
**Sex**	Birth records	Paediatric report	Paediatric report	Clinical records	Nurse assessment at birth and obstetric records
**Birth weight (g)**	Obstetric data, cohort study's own measures, birth records	Paediatric report	Paediatric report	Medical birth registry	Measured by midwives
**Gestational age (days)**	Derived from last menstrual period	Derived from last menstrual period or ultrasound data	Derived from last menstrual period	Derived from last menstrual period or ultrasound data or maternal report	Derived from an algorithm based on last menstrual period and ultrasound data
**Sibling position**	Questionnaire	Obstetrical report	Questionnaire	Questionnaire	Questionnaire
**Maternal age at birth (years)**	Calculated from maternal date of birth and reported delivery date	Calculated from maternal data of birth and birth date of the child	Calculated from maternal data of birth and birth date of the child	Medical birth registry	Obstetrics
**Mother born abroad**	Questionnaire	Questionnaire	Questionnaire	Questionnaire	Questionnaire
**Maternal education level when child was between 0 and <1 years**	Questionnaire	Questionnaire	Questionnaire	Questionnaire	Questionnaire
**Smoking in pregnancy**	Questionnaire	Questionnaire	Questionnaire	Questionnaire	Questionnaire
**Postpartum depression**	Edinburgh Postnatal Depression Scale	Edinburgh Postnatal Depression Scale	Edinburgh Postnatal Depression Scale	Not harmonized	Not harmonized
**EUSILC-based household income at baseline[a]**	Predicted	Predicted	Predicted	Predicted	Predicted
**Passive smoke exposure in the first year of life**	Questionnaire	Questionnaire	Questionnaire	Questionnaire	Questionnaire

[a]Log-equivalised total disposable household monthly income predicted using EUSILC data

Abbreviations: ALSPAC: Avon Longitudinal Study of Parents and Children; EDEN: Étude des Déterminants pré et postnatals du développement et de la santé de l'Enfant; ELFE: Étude Longitudinale Française depuis l'Enfance; INMA: INfancia y Medio Ambiente Project; SWS: Southampton Women's Survey

Table 3: Characteristics of the participating cohort population: Missing data and comparison of analyzed sample vs. excluded sample

	**ALSPAC (n=4847)**	Missings n (%)	**EDEN (n=1015)**	Missings n (%)	**ELFE (n=9100)**	Missings n (%)	**INMA (N=1348)**	Missings n (%)	**SWS (N=134)**	Missings n (%)	**Analyzed sample (n=16444)**	**Excluded sample (n=23938)**
**Child's characteristics**												
Sex, male, n (%)	2489 (51.4)	0 (0)	554 (54.6)	0 (0)	4693 (51.6)	0 (0)	682 (50.6)	0 (0)	78 (58.2)	0 (0)	8496 (51.7)	12260 (51.2)
Birth weight, grams, mean (SD)	3421 (539)	62 (1.3)	3310 (496)	0 (0)	3342 (478)	178 (2.0)	3258 (458)	3 (0.2)	3437 (560)	1 (0.8)	3357 (499)	3328 (561)
Gestational age, weeks, mean (SD)	39.9 (1.8)	9 (0.2)	39.7 (1.6)	0 (0)	39.6 (1.4)	126 (1.4)	39.9 (1.5)	0 (0)	39.5 (2.0)	0 (0)	39.7 (1.6)	39.6 (2.2)
First born, yes, n (%)	2034 (43.6)	181 (3.7)	484 (47.7)	0 (0)	4122 (45.9)	112 (1.2)	772 (57.5)	5 (0.4)	73 (54.5)	0 (0)	7485 (46.4)	9883 (45.8)
**Maternal characteristics**												
Maternal age at birth, years, mean (SD)	29.0 (4.6)	220 (4.5)	30.2 (4.6)	0 (0)	31.1 (4.5)	30 (0.3)	32.0 (4.0)	4 (0.3)	29.6 (3.4)	0 (0)	30.5 (4.6)	29.1 (5.1)
Mother born abroad, yes, n (%)	184 (4.3)	556 (11.5)	25 (2.5)	11 (1.1)	761 (8.4)	0 (0)	93 (6.9)	0 (0)	8 (6.0)	1 (0.8)	1071 (6.8)	2324 (11.7)
Maternal education level, n (%)												
High	694 (14.7)	121 (2.5)	628 (62.1)	3 (0.3)	6437 (70.8)	0 (0)	498 (37.0)	4 (0.3)	40 (29.9)	0 (0)	8297 (50.8)	6181 (28.9)
Middle	3291 (69.6)		344 (34.0)		2360 (25.9)		551 (41.0)		74 (55.2)		6620 (40.6)	11462 (53.7)
Low	741 (15.7)		40 (3.9)		303 (3.3)		295 (22.0)		20 (14.9)		1399 (8.6)	3724 (17.4)
Smoking during pregnancy, yes, n (%)	999 (22.7)	443 (9.1)	220 (21.7)	3 (0.3)	1418 (15.7)	82 (0.9)	393 (29.5)	17 (1.3)	21 (15.7)	0 (0)	3051 (19.2)	5866 (28.2)
Postpartum depression, yes, n (%)	380 (8.2)	206 (4.3)	74 (7.6)	45 (4.4)	756 (8.5)	159 (1.8)	NA	NA	NA	NA	1210 (8.3)	1387 (9.7)
**Household characteristics**												
EUSILC-based household income[a], mean (SD)	7.1 (0.2)	582 (12.0)	7.4 (0.3)	35 (3.5)	7.5 (0.3)	782 (8.6)	7.1 (0.3)	54 (4.0)	7.3 (0.3)	22 (16.4)	7.4 (0.3)	7.2 (0.3)
Passive smoke exposure in the 1st year of life, yes, n (%)	1601 (35.0)	277 (5.7)	420 (41.8)	10 (1.0)	3156 (35.2)	128 (1.4)	NA	NA	21 (15.7)	0 (0)	5198 (35.4)	6665 (38.6)
**Exposure**												
Sleep duration in hours:min, mean (SD)	11:30 (0:54)	0 (0)	12:36 (1:00)	0 (0)	12:18 (0:42)	0 (0)	10:24 (1:00)	0 (0)	11:24 (0:48)	0 (0)	11:54 (1:01)	11:38 (1:00)
Age at sleep duration measurement, years, mean (SD)	3.2 (0.1)	120 (2.5)	3.2 (0.1)	0 (0)	3.5 (0.2)	65 (0.7)	4.4 (0.2)	127 (9.4)	3.1 (0.1)	0 (0)	3.5 (0.3)	3.4 (0.3)

Table 3: Continued

	ALSPAC (n=4847)	Missings n (%)	EDEN (n=1015)	Missings n (%)	ELFE (n=9100)	Missings n (%)	INMA (N=1348)	Missin gs n (%)	SWS (N=134)	Missings n (%)	Analyzed sample (n=16444)	Excluded sample (n=23938)
**Outcomes**												
Internalizing behavior, percentile score[b], mean (SD)	42.5 (30.3)	0 (0)	50.0 (28.1)	0 (0)	42.8 (30.2)	0 (0)	NA	NA	NA	NA	43.6 (30.2)	46.3 (30.8)
Externalizing behavior, percentile score[b], mean (SD)	45.1 (29.3)	0 (0)	49.9 (28.6)	0 (0)	44.4 (29.3)	0 (0)	NA	NA	NA	NA	45.4 (29.4)	47.9 (30.0)
Age at behavior measurement, years, mean (SD)	4.1 (0.1)	0 (0)	5.6 (0.1)	0 (0)	5.5 (0.5)	0 (0)	NA	NA	NA	NA	5.1 (0.8)	5.2 (0.6)
Language standardized score[c], mean (SD)	101.0 (14.8)	0 (0)	100.0 (14.5)	0 (0)	NA	NA	100.0 (14.9)	0 (0)	99.0 (15.4)	0 (0)	100.2 (14.8)	99.1 (15.6)
Non-verbal intelligence standardized score[c], mean (SD)	101.4 (14.9)	0 (0)	100.6 (14.7)	0 (0)	NA	NA	100.3 (14.6)	0 (0)	99.6 (15.5)	0 (0)	100.3 (14.8)	98.0 (15.8)
Age at language/non-verbal intelligence measurement, years, mean (SD)	4.1 (0.03)	0 (0)	5.6 (0.1)	0 (0)	NA	NA	4.9 (0.6)	0 (0)	4.4 (0.1)	0 (0)	43.6 (30.2)	46.3 (30.8)

[a]Log-equivalised total disposable household income predicted using EUSILC data; [b]Measured with the SDQ in all cohorts; [c]Assessed by the WPPSI in ALSPAC, EDEN and SWS; assessed by the MSCA in INMA
Data are given as mean (standard deviation) or percentage (number). Sample sizes are based on children with data on sleep duration and on at least one outcome measure.
Abbreviations: ALSPAC: Avon Longitudinal Study of Parents and Children; EDEN: Étude des Déterminants pré et postnatals du développement et de la santé de l'Enfant; ELFE: Étude Longitudinale Française depuis l'Enfance; INMA: INfancia y Medio Ambiente Project; NA= not available or not harmonized by the specific cohort; SWS: Southampton Women's Survey

Table 4: Association between total sleep duration per day at mean age of 3.5 years and internalizing/externalizing behavior (percentile and raw score) at mean age of 5.1 years using one-stage IPD meta-analysis – basic models

Outcome	N	Estimate (95%-CI)
Internalizing behavior percentile score	11920	-1.25 (-1.93, -0.57)
Internalizing behavior raw score	11854	-0.10 (-0.15, -0.04)
Externalizing behavior percentile score	11920	-2.29 (-2.94, -1.64)
Externalizing behavior raw score	11859	-0.27 (-0.35, -0.20)

Basic model adjusted for age at outcome measurement, sex of the child and cohort
Abbreviations: N: Number of children included in the analysis; 95% CI: 95% confidence interval

Table 5: Association between total sleep duration per day at mean age of 3.5 years and internalizing/externalizing behavior (percentile and raw score) at mean age of 5.1 years using one-stage IPD meta-analysis – adjusted models

Adjusted model

Outcome	N	Estimate (95%-CI)
Internalizing behavior percentile score	11920	-1.18 (-1.85, -0.51)
Internalizing behavior raw score	11854	-0.09 (-0.15, -0.04)
Externalizing behavior percentile score	11920	-2.39 (-3.04, -1.75)
Externalizing behavior raw score	11859	-0.29 (-0.36, -0.21)

Adjusted for sex of the child, age at outcome measurement, cohort, mother's age at birth, maternal education, postpartum depression, mother born abroad, birthweight, gestational age, sibling position, passive smoke exposure in the first year of life, EUSILC-based household income
Abbreviations: N: Number of children included in the analysis; 95% CI: 95% confidence interval

Additionally adjusted for TV-watching during preschool age

Outcome	N	Estimate (95%-CI)
Internalizing behavior percentile score	11687	-0.96 (-1.64, -0.27)
Internalizing behavior raw score	11632	-0.08 (-0.14, -0.02)
Externalizing behavior percentile score	11688	-2.12 (-2.78, -1.47)
Externalizing behavior raw score	11631	-0.25 (-0.33, -0.18)

Adjusted for sex of the child, age at outcome measurement, cohort, mother's age at birth, maternal education, postpartum depression, mother born abroad, birthweight, gestational age, sibling position, passive smoke exposure in the first year of life, EUSILC-based household income, TV-watching
Abbreviations: N: Number of children included in the analysis; 95% CI: 95% confidence interval

Additionally adjusted for child attending a daycare centre

Outcome	N	Estimate (95%-CI)
Internalizing behavior percentile score	11588	-0.91 (-1.60, -0.22)
Internalizing behavior raw score	11535	-0.08 (-0.13, -0.02)
Externalizing behavior percentile score	11589	-2.10 (-2.76, -1.44)
Externalizing behavior raw score	11533	-0.25 (-0.33, -0.18)

Adjusted for sex of the child, age at outcome measurement, cohort, mother's age at birth, maternal education, postpartum depression, mother born abroad, birthweight, gestational age, sibling position, passive smoke exposure in the first year of life, EUSILC-based household income, TV-watching, child attending a daycare centre in the first 4 years of life
Abbreviations: N: Number of children included in the analysis; 95% CI: 95% confidence interval

Table 6: Association between total sleep duration per day at mean age of 3.7 years and language/non-verbal intelligence (standardized score) at mean age of 4.9 years using one-stage IPD meta-analysis – basic models

Outcome	N	Estimate (95%-CI)
Language standardized score	2979	-0.37 (-0.95, 0.20)
Non-verbal intelligence standardized score	2981	-0.53 (-1.10, -0.04)

Basic model adjusted for age at outcome measurement, sex of the child and cohort
Abbreviations: N: Number of children included in the analysis; 95% CI: 95% confidence interval

Table 7: Association between total sleep duration per day at mean age of 3.7 years and language/non-verbal intelligence (standardized score) at mean age of 4.9 years using one-stage IPD meta-analysis – adjusted models

Adjusted model

Outcome	N	Estimate (95%-CI)
Language standardized score	2979	-0.26 (-0.81, 0.28)
Non-verbal intelligence standardized score	2981	-0.46 (-1.01, 0.09)

Adjusted for sex of the child, age at outcome measurement, cohort, mother's age at birth, maternal education, mother born abroad, birthweight, gestational age, sibling position, smoking during pregnancy, EUSILC-based household income
Abbreviations: N: Number of children included in the analysis; 95% CI: 95% confidence interval

Additionally adjusted for TV-watching during preschool age

Outcome	N	Estimate (95%-CI)
Language standardized score	2857	-0.20 (-0.76, 0.36)
Non-verbal intelligence standardized score	2859	-0.42 (-0.98, 0.15)

Adjusted for sex of the child, age at outcome measurement, cohort, mother's age at birth, maternal education, mother born abroad, birthweight, gestational age, sibling position, smoking during pregnancy, EUSILC-based household income, TV-watching
Abbreviations: N: Number of children included in the analysis; 95% CI: 95% confidence interval

Additionally adjusted for child attending a daycare centre

Outcome	N	Estimate (95%-CI)
Language standardized score	2760	-0.22 (-0.79, 0.35)
Non-verbal intelligence standardized score	2762	-0.51 (-1.08, 0.06)

Adjusted for sex of the child, age at outcome measurement, cohort, mother's age at birth, maternal education, mother born abroad, birthweight, gestational age, sibling position, smoking during pregnancy, EUSILC-based household income, TV-watching, child attending a daycare centre in the first 4 years of life
Abbreviations: N: Number of children included in the analysis; 95% CI: 95% confidence interval

Table 8: Associations between sleep tertiles and internalizing behavior, externalizing behavior, language and non-verbal intelligence using two-stage IPD meta-analysis – basic models

	**Internalizing percentile score**	**Externalizing percentile score**	**Language standardized score**	**Non-verbal intelligence standardized score**
Number of cohorts (N)	3 (11920)	3 (11920)	4 (2979)	4 (2981)
	**Estimate [95% CI]**			
1st tertile	1.68 [-0.57, 3.93]	2.64 [1.37, 3.91]	-0.68 [-3.99, 2.63]	0.07 [-1.49, 1.63]
2nd tertile	ref.	ref.	ref.	ref.
3rd tertile	0.29 [-2.42, 3.00]	-1.19 [-2.46, 0.08]	-0.66 [-2.50, 1.18]	-1.37 [-2.65, -0.08]

All models adjusted for sex of the child and age at outcome measurement
Abbreviations: N: Number of children included in the analysis; 95% CI: 95% confidence interval

Table 9: Associations between sleep tertiles and internalizing behavior, externalizing behavior, language and non-verbal intelligence using two-stage IPD meta-analysis – adjusted models

We used the 2nd third of sleep duration as reference to investigate the possibility that both shorter and longer sleep duration might be associated with the outcome (non-linear association). Children from the 1st sleep duration third group (children in the lowest third of sleep duration), had a higher externalizing behavior percentile score than children in the middle third and those in the highest third had a lower score than the middle third, thus having consistency with a monotonic relationship of lower score with longer sleep duration. There was no difference in sleep duration thirds for internalizing behavior, language or non-verbal intelligence scores. All in all, there was no evidence for a non-linear association between sleep duration and behavioral or cognitive outcomes.

	**Internalizing percentile score**[a]	**Externalizing percentile score**[a]	**Language standardized score**[b]	**Non-verbal intelligence standardized score**[b]
Number of cohorts (N)	3 (11920)	3 (11920)	4 (2979)	4 (2981)
	**Estimate [95% CI]**			
1st third	1.28 [-0.81, 3.38]	2.56 [1.31, 3.81]	-0.39 [-2.91, 2.13]	0.11 [-1.14, 1.36]
2nd third	ref.	ref.	ref.	ref.
3rd third	-0.24 [-2.44, 1.96]	-1.56 [-2.82, -0.31]	-0.22 [-1.88, 1.43]	-0.94 [-2.18, 0.31]

[a]Adjusted for sex of the child, age at outcome measurement, maternal age at birth, maternal education, postpartum depression, mother born abroad, birthweight, gestational age, siblings position, passive smoke exposure in the first year of life, EUSILC-based household income
[b]Adjusted for sex of the child, age at outcome measurement, maternal age at birth, maternal education, mother born abroad, birthweight, gestational age, siblings position, smoking in pregnancy, EUSILC-based household income
Abbreviations: N: Number of children included in the analysis; 95% CI: 95% confidence interval

Figures

Figure 1: Directed acyclic graphs illustrating the variables required to control for confounding between sleep duration and 1a) problem behavior, 1b) cognitive outcomes

The green circle with the triangle is the exposure (sleep duration) and the blue circle with the "I" is the outcome (problem behavior/cognitive outcomes). Potential confounders are illustrated by red circles. Variables that are not expected to influence the outcome but are related to the exposure are illustrated by green circles. Variables that are not expected to influence the exposure but are related to the outcome are illustrated by blue circles. Arrows show the direction of causal association.

a: Sleep duration and problem behavior

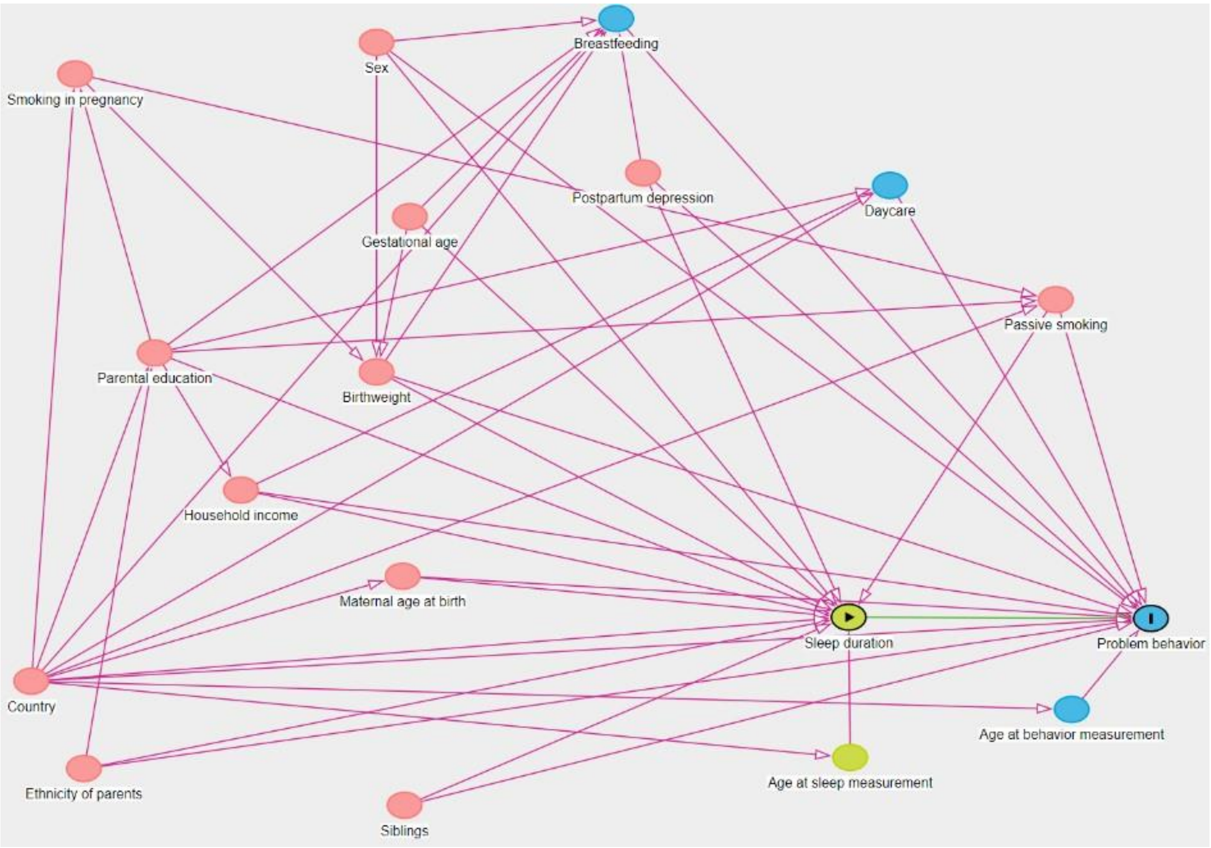

b: Sleep duration and cognitive outcomes

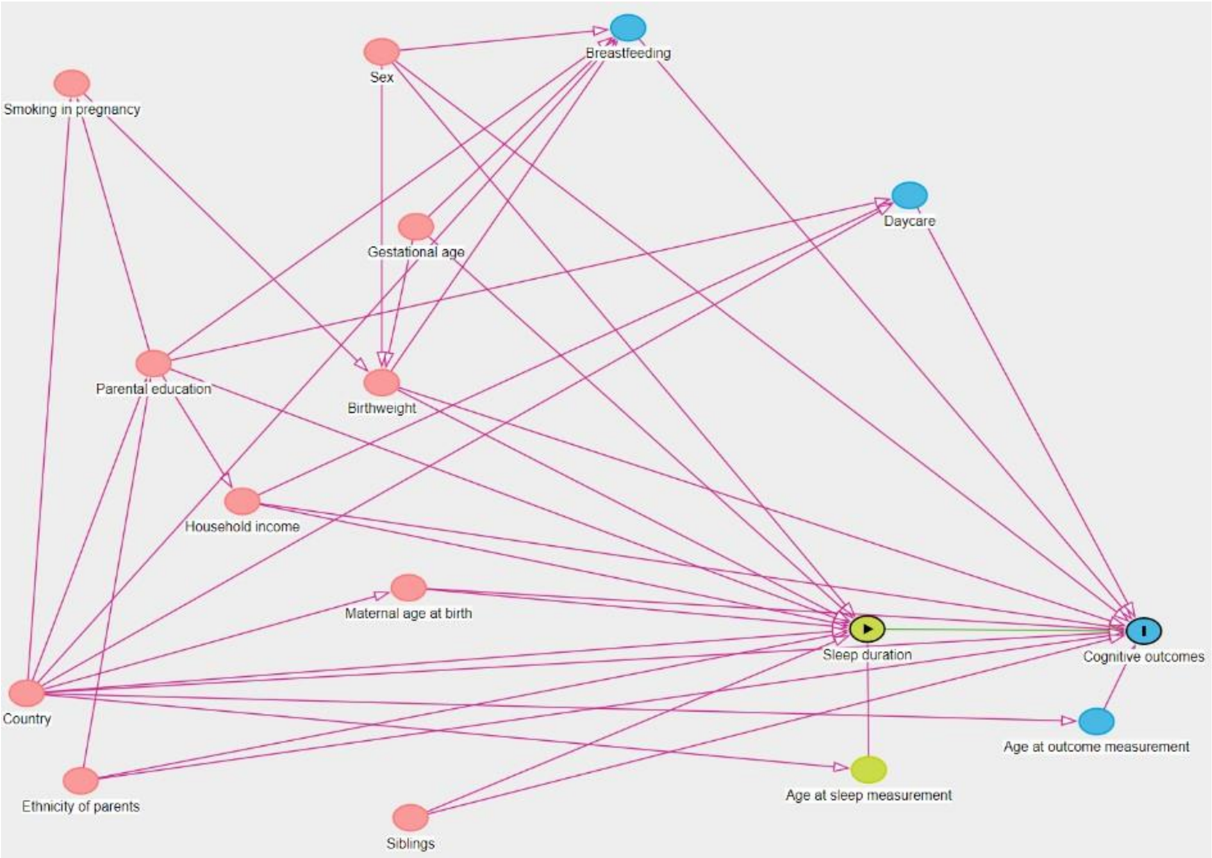

Figure 2: Association between total sleep duration per day at mean age of 3.5 years and 2a) internalizing behavior (raw score), 2b) externalizing behavior (raw score) at mean age of 5.1 years using two-stage IPD meta-analysis – basic models

a: Internalizing behavior (raw score) **b: Externalizing behavior (raw score)**

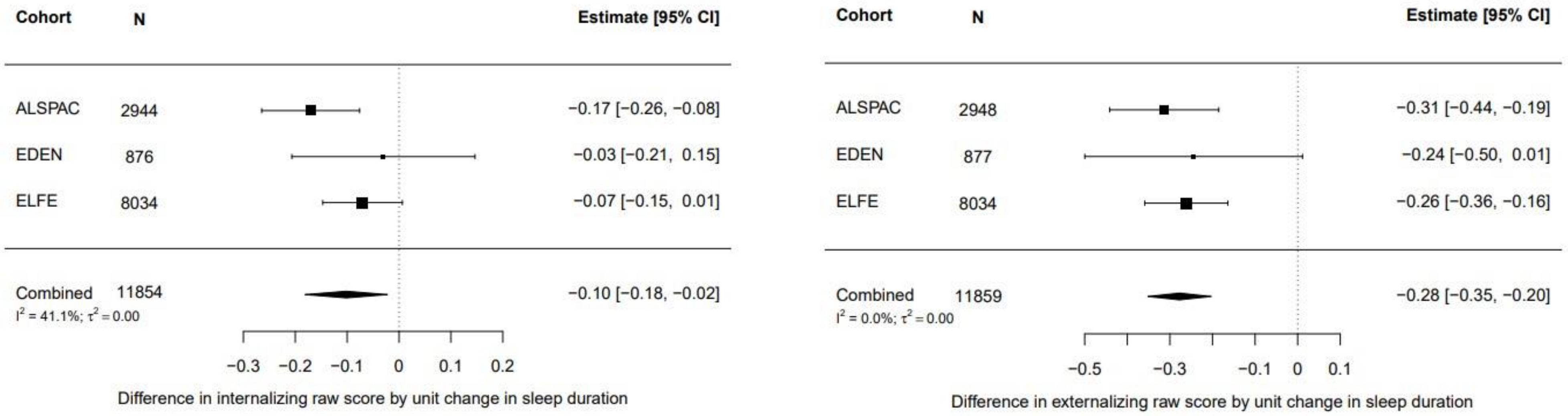

Basic model adjusted for age at outcome measurement and sex of the child

Abbreviations: ALSPAC: Avon Longitudinal Study of Parents and Children, CI: Confidence interval, EDEN: Étude des Déterminants pré et postnatals du développement et de la santé de l'Enfant, ELFE: Étude Longitudinale Française depuis l'Enfance, N: Number of children included in the analysis; I^2 and τ^2 statistics represent between cohort heterogeneity

Figure 3: Association between total sleep duration per day at mean age of 3.5 years and 3a) internalizing behavior (percentile score), 3b) externalizing behavior (percentile score) at mean age of 5.1 years using two-stage IPD meta-analysis – basic models

a: Internalizing behavior (percentile score)

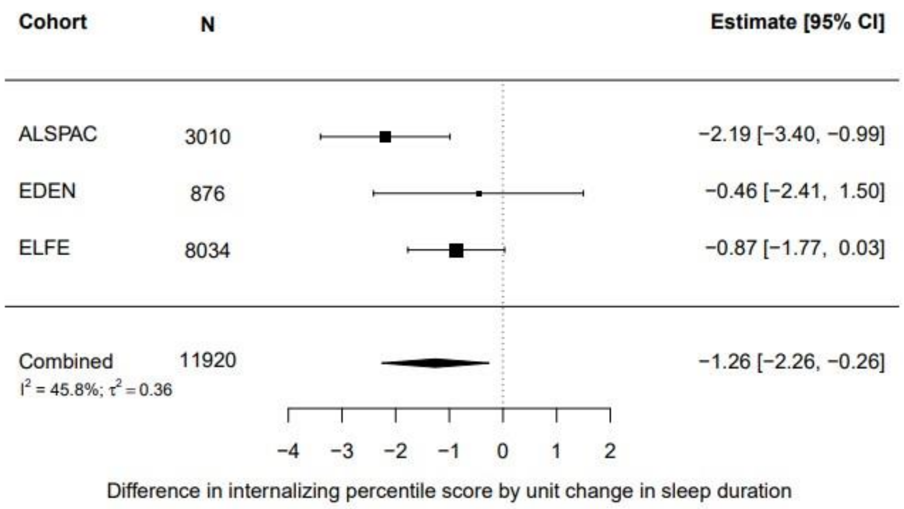

b: Externalizing behavior (percentile score)

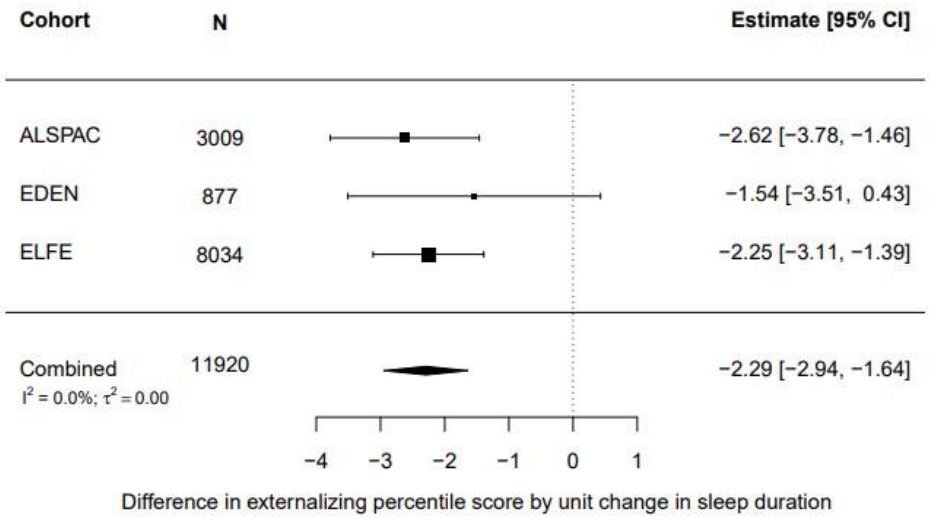

Basic model adjusted for age at outcome measurement and sex of the child

Abbreviations: ALSPAC: Avon Longitudinal Study of Parents and Children, CI: Confidence interval, EDEN: Étude des Déterminants pré et postnatals du développement et de la santé de l'Enfant, ELFE: Étude Longitudinale Française depuis l'Enfance, N: Number of children included in the analysis; I^2 and τ^2 statistics represent between cohort heterogeneity

Figure 4: Association between total sleep duration per day at mean age of 3.5 years and internalizing behavior (raw score) at mean age of 5.1 years using two-stage IPD meta-analysis – adjusted models

Adjusted model

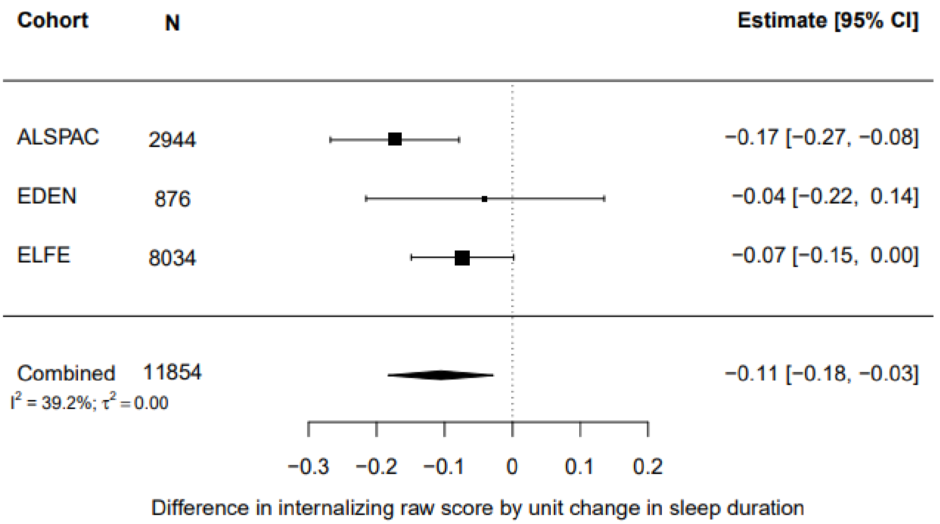

Adjusted for sex of the child, age at outcome measurement, mother's age at birth, maternal education, postpartum depression, mother born abroad, birthweight, gestational age, sibling position, passive smoke exposure in the first year of life, EUSILC-based household income
Abbreviations: ALSPAC: Avon Longitudinal Study of Parents and Children, CI: Confidence interval, EDEN: Étude des Déterminants pré et postnatals du développement et de la santé de l'Enfant, ELFE: Étude Longitudinale Française depuis l'Enfance, N: Number of children included in the analysis; I^2 and τ^2 statistics represent between cohort heterogeneity

Additionally adjusted for TV-watching during preschool age

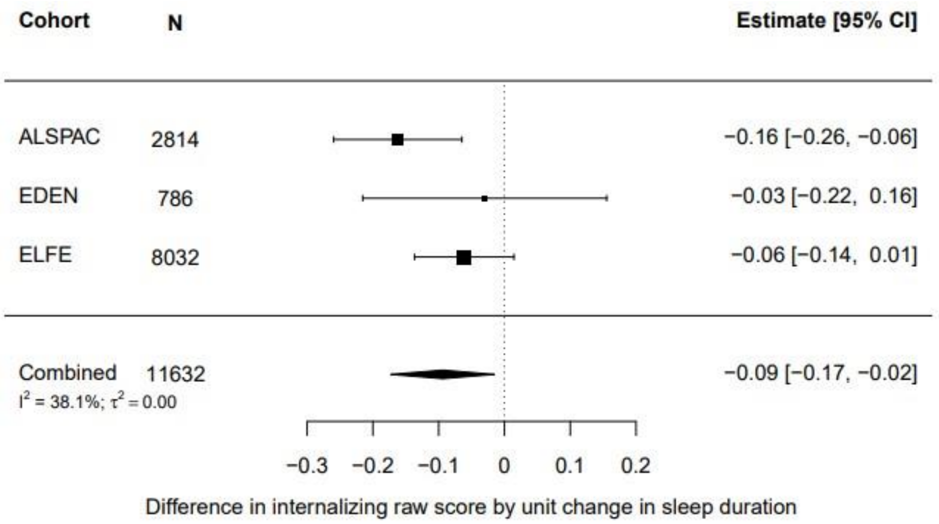

Adjusted for sex of the child, age at outcome measurement, mother's age at birth, maternal education, postpartum depression, mother born abroad, birthweight, gestational age, sibling position, passive smoke exposure in the first year of life, EUSILC-based household income, TV-watching
Abbreviations: ALSPAC: Avon Longitudinal Study of Parents and Children, CI: Confidence interval, EDEN: Étude des Déterminants pré et postnatals du développement et de la santé de l'Enfant, ELFE: Étude Longitudinale Française depuis l'Enfance, N: Number of children included in the analysis; I^2 and τ^2 statistics represent between cohort heterogeneity

Additionally adjusted for child attending a daycare centre

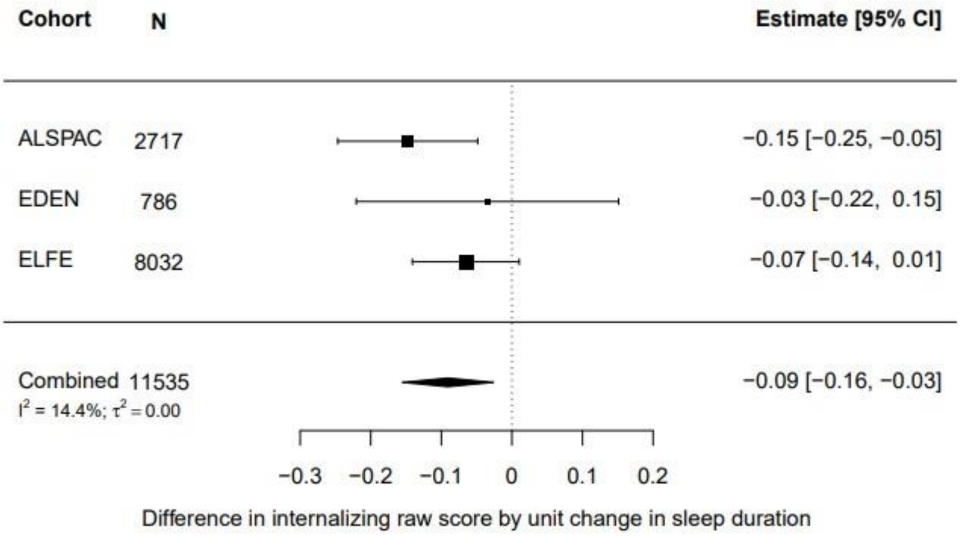

Adjusted for sex of the child, age at outcome measurement, mother's age at birth, maternal education, postpartum depression, mother born abroad, birthweight, gestational age, sibling position, passive smoke exposure in the first year of life, EUSILC-based household income, TV-watching, child attending a daycare centre in the first 4 years of life

Abbreviations: ALSPAC: Avon Longitudinal Study of Parents and Children, CI: Confidence interval, EDEN: Étude des Déterminants pré et postnatals du développement et de la santé de l'Enfant, ELFE: Étude Longitudinale Française depuis l'Enfance, N: Number of children included in the analysis; I^2 and τ^2 statistics represent between cohort heterogeneity

Figure 5: Association between total sleep duration per day at mean age of 3.5 years and internalizing behavior (percentile score) at mean age of 5.1 years using two-stage IPD meta-analysis – adjusted models

Additionally adjusted for TV-watching during preschool age

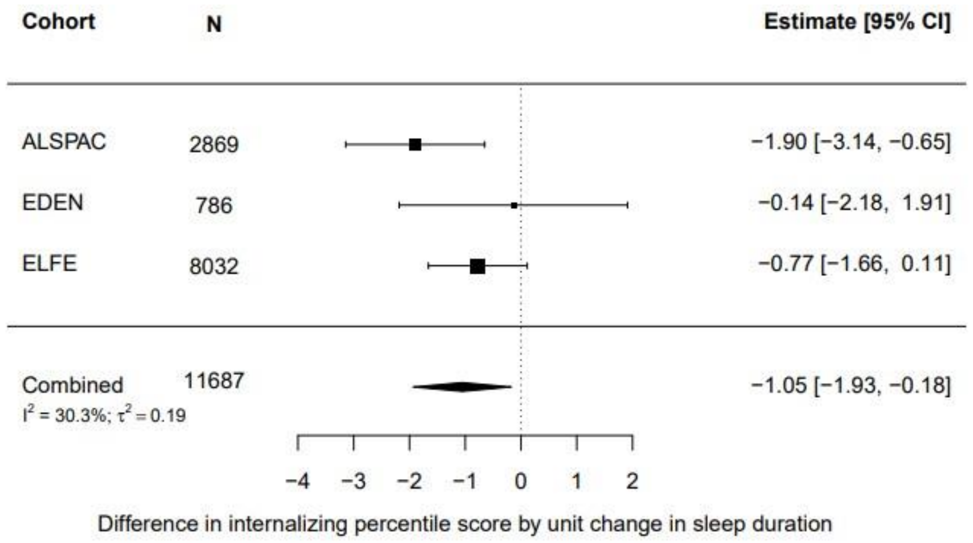

Adjusted for sex of the child, age at outcome measurement, mother's age at birth, maternal education, postpartum depression, mother born abroad, birthweight, gestational age, sibling position, passive smoke exposure in the first year of life, EUSILC-based household income, TV-watching

Abbreviations: ALSPAC: Avon Longitudinal Study of Parents and Children, CI: Confidence interval, EDEN: Étude des Déterminants pré et postnatals du développement et de la santé de l'Enfant, ELFE: Étude Longitudinale Française depuis l'Enfance, N: Number of children included in the analysis; I^2 and τ^2 statistics represent between cohort heterogeneity

Additionally adjusted for child attending a daycare centre

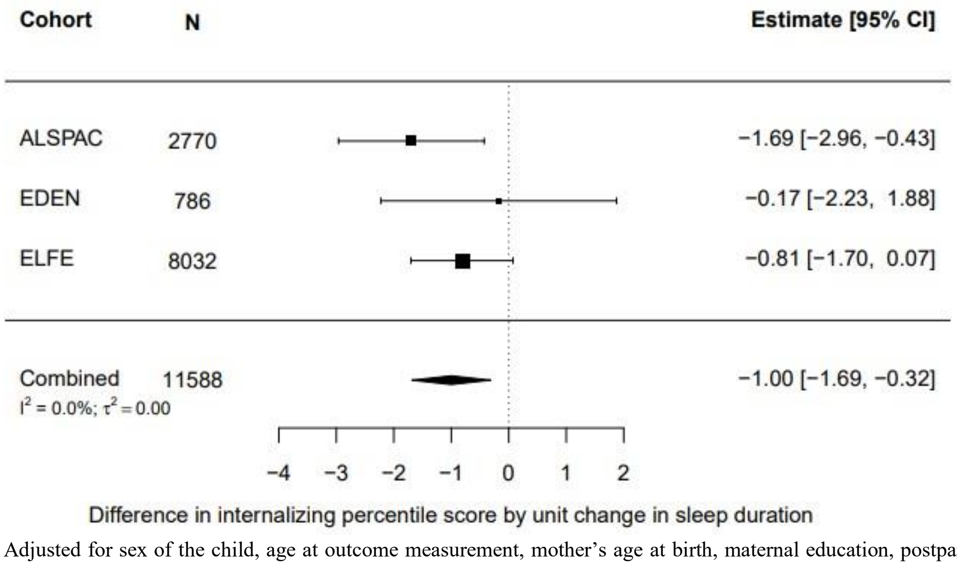

Adjusted for sex of the child, age at outcome measurement, mother's age at birth, maternal education, postpartum depression, mother born abroad, birthweight, gestational age, sibling position, passive smoke exposure in the first year of life, EUSILC-based household income, TV-watching, child attending a daycare centre in the first 4 years of life

Abbreviations: ALSPAC: Avon Longitudinal Study of Parents and Children, CI: Confidence interval, EDEN: Étude des Déterminants pré et postnatals du développement et de la santé de l'Enfant, ELFE: Étude Longitudinale Française depuis l'Enfance, N: Number of children included in the analysis; I^2 and τ^2 statistics represent between cohort heterogeneity

Figure 6: Association between total sleep duration per day at mean age of 3.5 years and externalizing behavior (raw score) at mean age of 5.1 years using two-stage IPD meta-analysis – adjusted models

Adjusted model

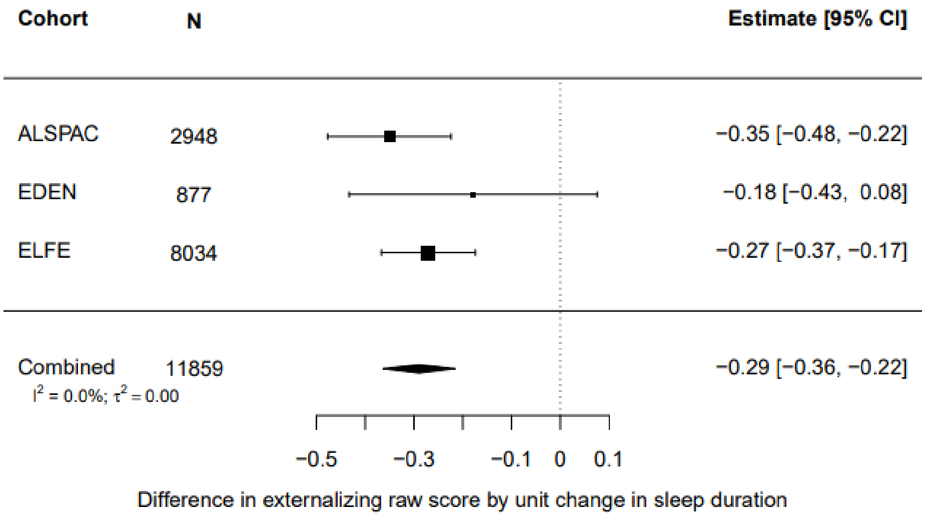

Adjusted for sex of the child, age at outcome measurement, mother's age at birth, maternal education, postpartum depression, mother born abroad, birthweight, gestational age, sibling position, passive smoke exposure in the first year of life, EUSILC-based household income

Abbreviations: ALSPAC: Avon Longitudinal Study of Parents and Children, CI: Confidence interval, EDEN: Étude des Déterminants pré et postnatals du développement et de la santé de l'Enfant, ELFE: Étude Longitudinale Française depuis l'Enfance, N: Number of children included in the analysis; I^2 and τ^2 statistics represent between cohort heterogeneity

Additionally adjusted for TV-watching during preschool age

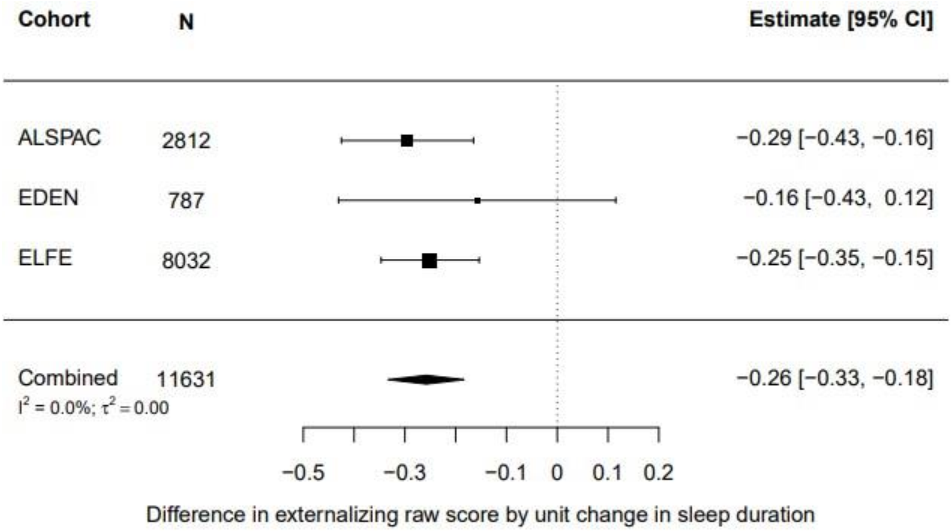

Adjusted for sex of the child, age at outcome measurement, mother's age at birth, maternal education, postpartum depression, mother born abroad, birthweight, gestational age, sibling position, passive smoke exposure in the first year of life, EUSILC-based household income, TV-watching

Abbreviations: ALSPAC: Avon Longitudinal Study of Parents and Children, CI: Confidence interval, EDEN: Étude des Déterminants pré et postnatals du développement et de la santé de l'Enfant, ELFE: Étude Longitudinale Française depuis l'Enfance, N: Number of children included in the analysis; I^2 and τ^2 statistics represent between cohort heterogeneity

Additionally adjusted for child attending a daycare centre

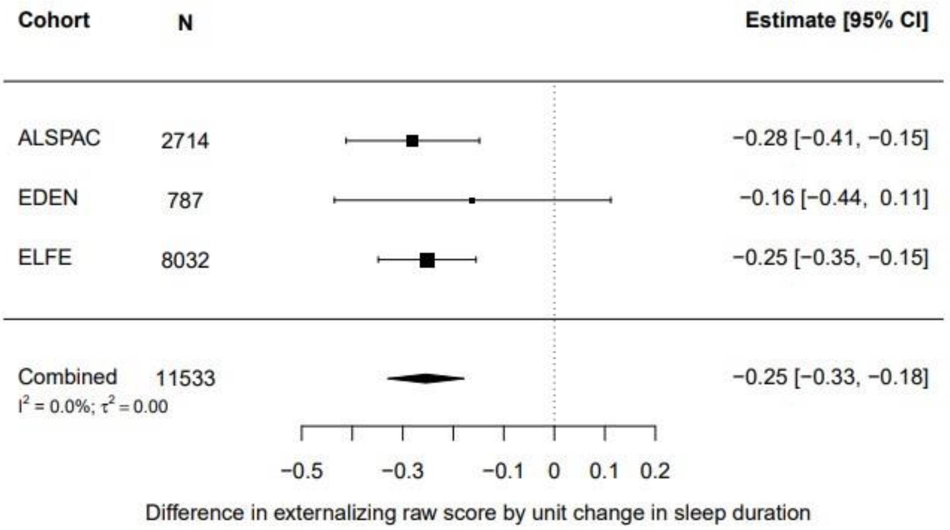

Adjusted for sex of the child, age at outcome measurement, mother's age at birth, maternal education, postpartum depression, mother born abroad, birthweight, gestational age, sibling position, passive smoke exposure in the first year of life, EUSILC-based household income, TV-watching, child attending a daycare centre in the first 4 years of life

Abbreviations: ALSPAC: Avon Longitudinal Study of Parents and Children, CI: Confidence interval, EDEN: Étude des Déterminants pré et postnatals du développement et de la santé de l'Enfant, ELFE: Étude Longitudinale Française depuis l'Enfance, N: Number of children included in the analysis; I^2 and τ^2 statistics represent between cohort heterogeneity

Figure 7: Association between total sleep duration per day at mean age of 3.5 years and externalizing behavior (percentile score) at mean age of 5.1 years using two-stage IPD meta-analysis – adjusted models

Additionally adjusted for TV-watching during preschool age

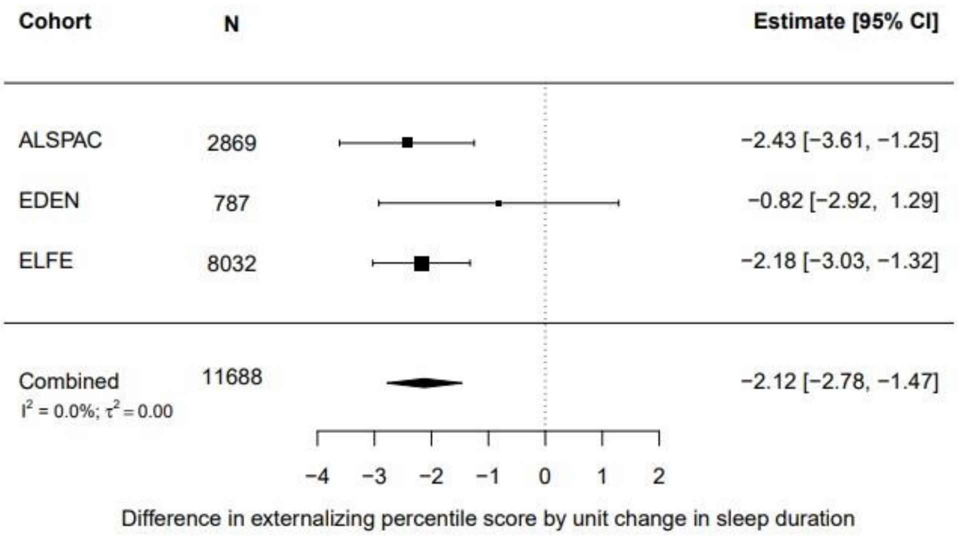

Adjusted for sex of the child, age at outcome measurement, mother's age at birth, maternal education, postpartum depression, mother born abroad, birthweight, gestational age, sibling position, passive smoke exposure in the first year of life, EUSILC-based household income, TV-watching

Abbreviations: ALSPAC: Avon Longitudinal Study of Parents and Children, CI: Confidence interval, EDEN: Étude des Déterminants pré et postnatals du développement et de la santé de l'Enfant, ELFE: Étude Longitudinale Française depuis l'Enfance, N: Number of children included in the analysis; I^2 and τ^2 statistics represent between cohort heterogeneity

Additionally adjusted for child attending a daycare centre

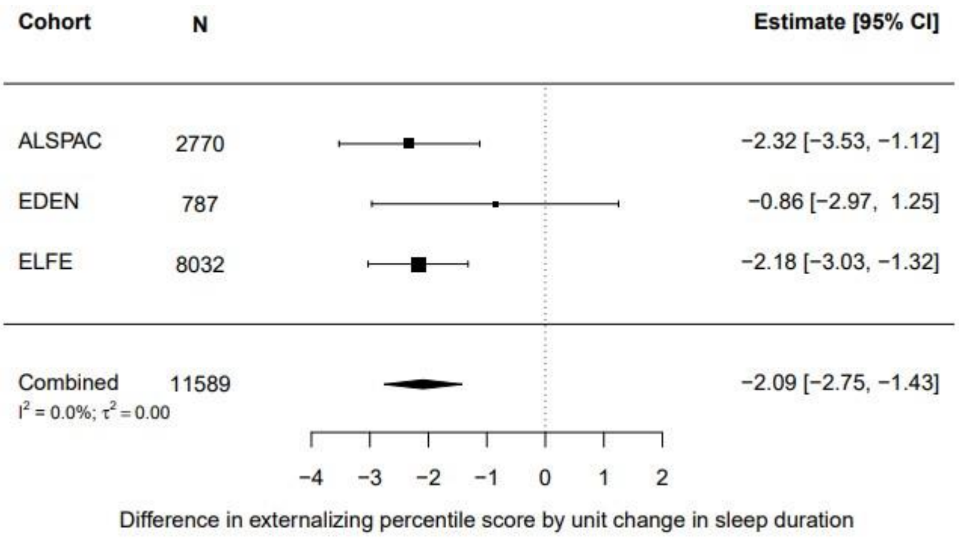

Adjusted for sex of the child, age at outcome measurement, mother's age at birth, maternal education, postpartum depression, mother born abroad, birthweight, gestational age, sibling position, passive smoke exposure in the first year of life, EUSILC-based household income, TV-watching, child attending a daycare centre in the first 4 years of life

Abbreviations: ALSPAC: Avon Longitudinal Study of Parents and Children, CI: Confidence interval, EDEN: Étude des Déterminants pré et postnatals du développement et de la santé de l'Enfant, ELFE: Étude Longitudinale Française depuis l'Enfance, N: Number of children included in the analysis; I^2 and τ^2 statistics represent between cohort heterogeneity

Figure 8: Association between total sleep duration per day at mean age of 3.5 years and 8a) internalizing behavior (percentile score), 8b) externalizing behavior (percentile score) at mean age of 5.1 years using two-stage IPD meta-analysis: Twins and children with congenital malformation, cerebral palsy excluded from analysis – adjusted models

a: Internalizing behavior (percentile score) **b: Externalizing behavior (percentile score)**

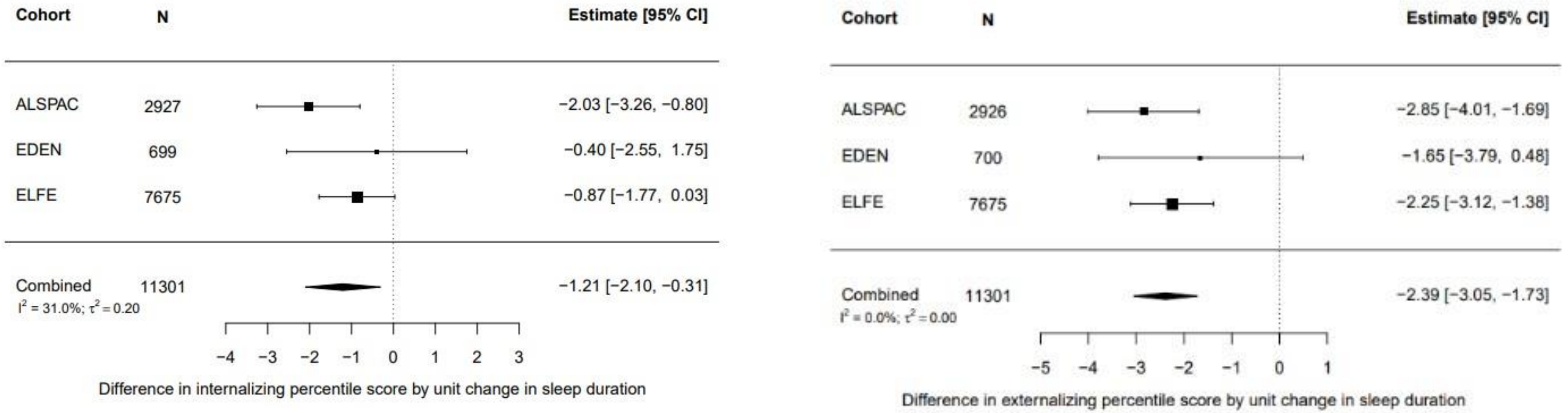

Adjusted for sex of the child, age at outcome measurement, mother's age at birth, maternal education, postpartum depression, mother born abroad, birthweight, gestational age, sibling position, passive smoke exposure in the first year of life, EUSILC-based household income

In the ELFE and ALSPAC cohort twins were included, in the EDEN cohort twins were an exclusion criteria. In the EDEN and ELFE cohort children with congenital anomalies were included, in the ALSPAC cohort it was an exclusion criteria. In the ELFE cohort children with cerebral palsy were included, in the ALSPAC and EDEN cohort it was an exclusion criteria.

Abbreviations: ALSPAC: Avon Longitudinal Study of Parents and Children, CI: Confidence interval, EDEN: Étude des Déterminants pré et postnatals du développement et de la santé de l'Enfant, ELFE: Étude Longitudinale Française depuis l'Enfance, N: Number of children included in the analysis; I^2 and τ^2 statistics represent between cohort heterogeneity

Figure 9: Association between total sleep duration per day at mean age of 3.7 years and 9a) language (standardized score), 9b) non-verbal intelligence (standardized score) at mean age of 4.9 years using two-stage IPD meta-analysis – basic models

a: Language (standardized score)

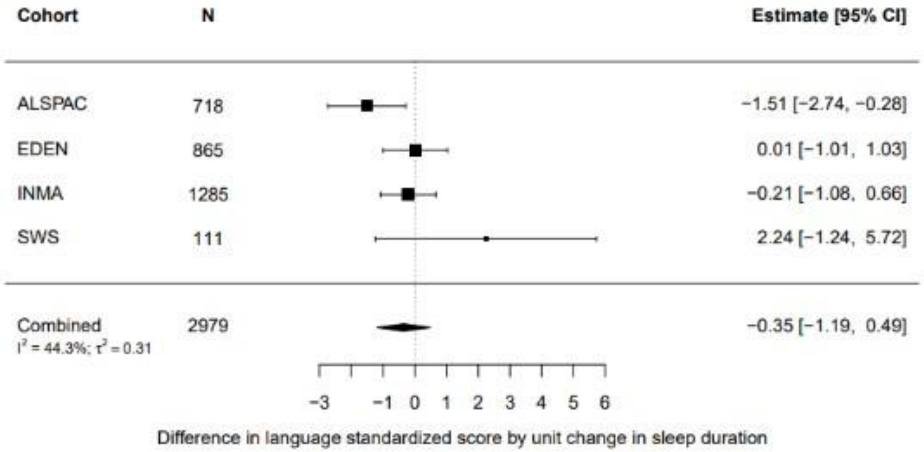

b: Non-verbal intelligence (standardized score)

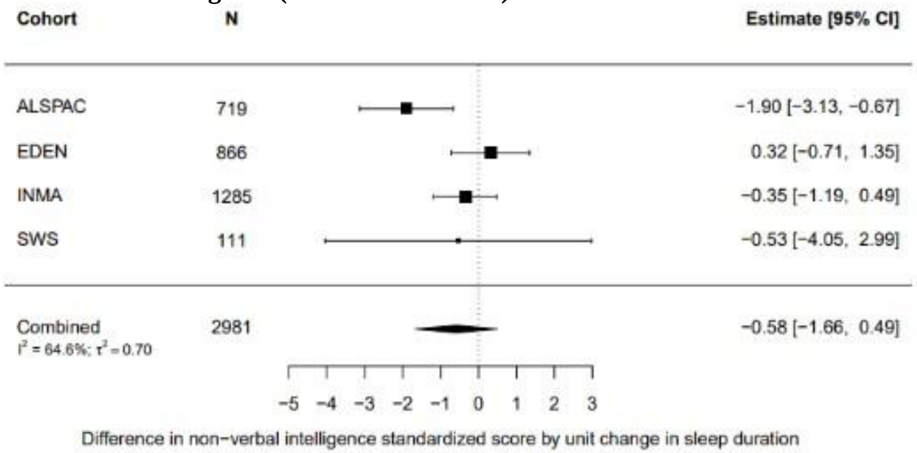

Basic model adjusted for age at outcome measurement and sex of the child

Abbreviations: ALSPAC: Avon Longitudinal Study of Parents and Children, CI: Confidence interval, EDEN: Étude des Déterminants pré et postnatals du développement et de la santé de l'Enfant, INMA: INfancia y Medio Ambiente Project, SWS: Southampton Women's Survey, N: Number of children included in the analysis; I^2 and τ^2 statistics represent between cohort heterogeneity

Figure 10: Association between total sleep duration per day at mean age of 3.7 years and language (standardized score) at mean age of 4.9 years using two-stage IPD meta-analysis – adjusted models

Additionally adjusted for TV-watching during preschool age

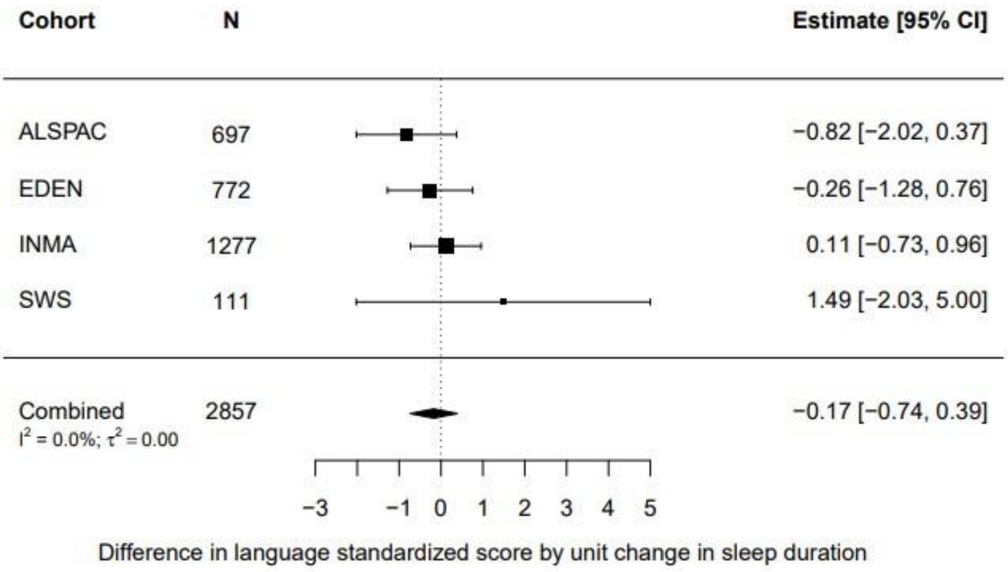

Adjusted for sex of the child, age at outcome measurement, mother's age at birth, maternal education, maternal country of birth, birthweight, gestational age, sibling position, smoking in pregnancy, EUSILC-based household income, TV-watching

Abbreviations: ALSPAC: Avon Longitudinal Study of Parents and Children, CI: Confidence interval, EDEN: Étude des Déterminants pré et postnatals du développement et de la santé de l'Enfant, INMA: INfancia y Medio Ambiente Project, SWS: Southampton Women's Survey, N: Number of children included in the analysis; I^2 and τ^2 statistics represent between cohort heterogeneity

Additionally adjusted for child attending a daycare centre

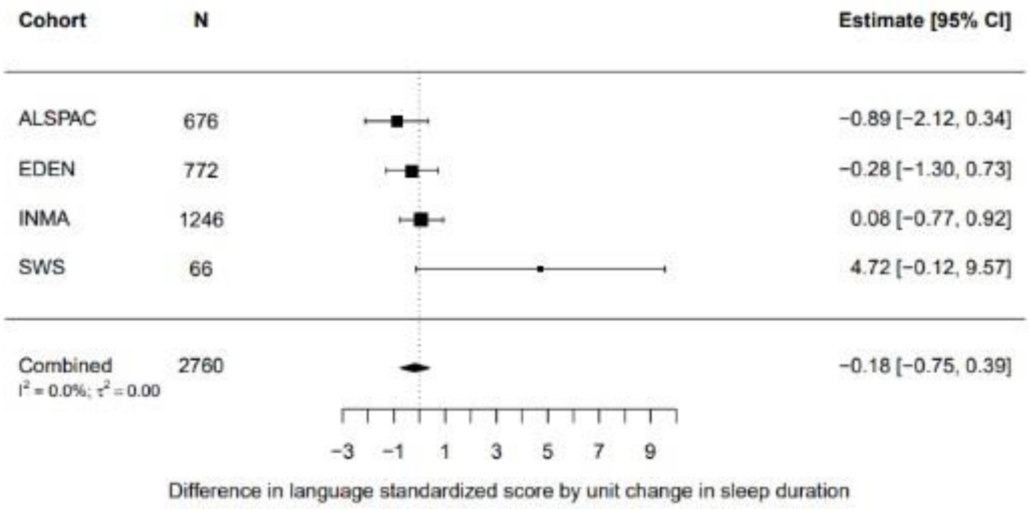

Adjusted for sex of the child, age at outcome measurement, mother's age at birth, maternal education, maternal country of birth, birthweight, gestational age, sibling position, smoking in pregnancy, EUSILC-based household income, TV-watching, child attending a daycare center in the first 4 years of life

Abbreviations: ALSPAC: Avon Longitudinal Study of Parents and Children, CI: Confidence interval, EDEN: Étude des Déterminants pré et postnatals du développement et de la santé de l'Enfant, INMA: INfancia y Medio Ambiente Project, SWS: Southampton Women's Survey, N: Number of children included in the analysis; I^2 and τ^2 statistics represent between cohort heterogeneity

Figure 11: Association between total sleep duration per day at mean age of 3.7 years and non-verbal intelligence (standardized score) at mean age of 4.9 years using two-stage IPD meta-analysis – adjusted models

Additionally adjusted for TV-watching during preschool age

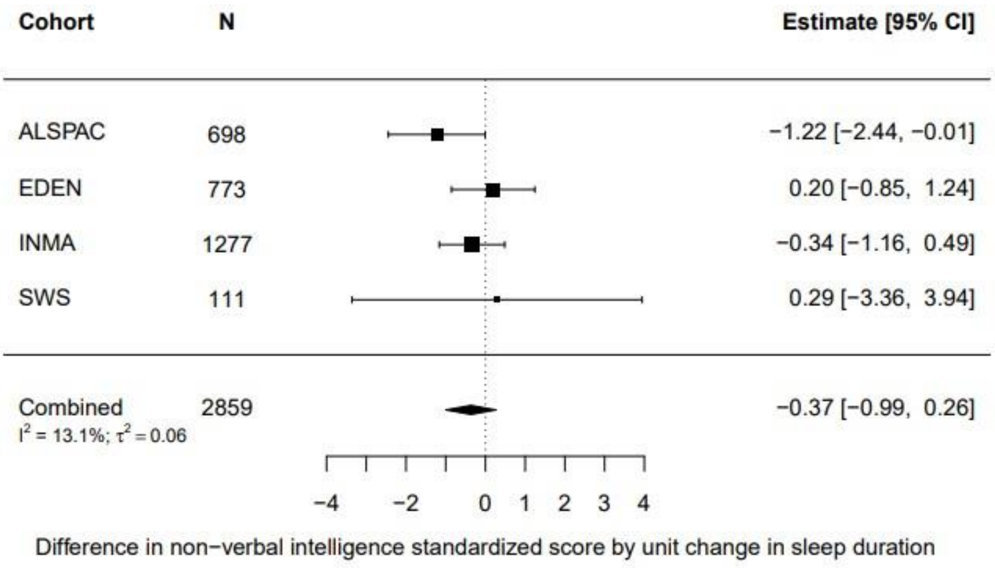

Adjusted for sex of the child, age at outcome measurement, mother's age at birth, maternal education, maternal country of birth, birthweight, gestational age, sibling position, smoking in pregnancy, EUSILC-based household income, TV-watching

Abbreviations: ALSPAC: Avon Longitudinal Study of Parents and Children, CI: Confidence interval, EDEN: Étude des Déterminants pré et postnatals du développement et de la santé de l'Enfant, INMA: INfancia y Medio Ambiente Project, SWS: Southampton Women's Survey, N: Number of children included in the analysis; I^2 and τ^2 statistics represent between cohort heterogeneity

Additionally adjusted for child attending a daycare centre

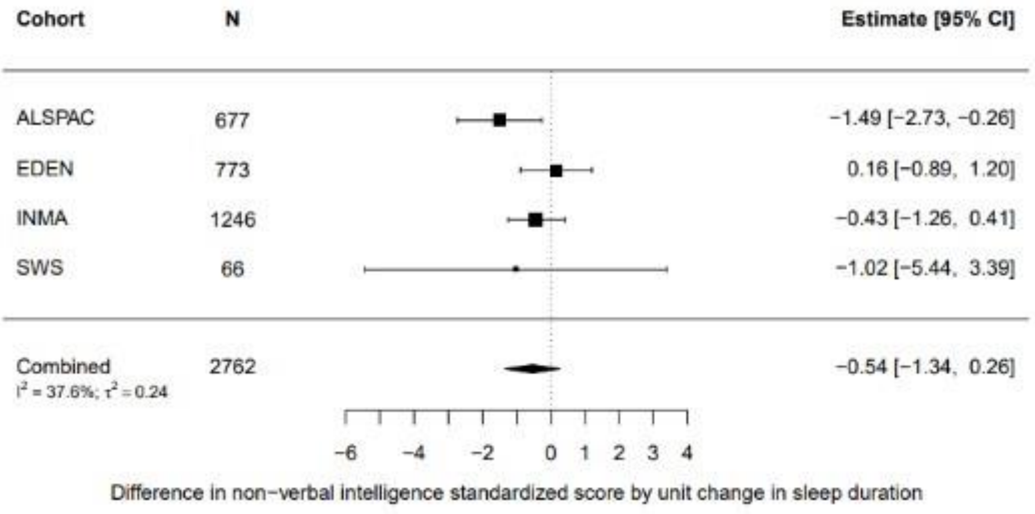

Adjusted for sex of the child, age at outcome measurement, mother's age at birth, maternal education, maternal country of birth, birthweight, gestational age, sibling position, smoking in pregnancy, EUSILC-based household income, TV-watching, child attending a daycare center in the first 4 years of life

Abbreviations: ALSPAC: Avon Longitudinal Study of Parents and Children, CI: Confidence interval, EDEN: Étude des Déterminants pré et postnatals du développement et de la santé de l'Enfant, INMA: INfancia y Medio Ambiente Project, SWS: Southampton Women's Survey, N: Number of children included in the analysis; I^2 and τ^2 statistics represent between cohort heterogeneity

Figure 12: Association between total sleep duration per day at mean age of 3.7 years and 12a) language (standardized score), 12b) non-verbal intelligence (standardized score) at mean age of 4.9 years using two-stage IPD meta-analysis: Twins and children with congenital malformation excluded from analysis – adjusted models

a: Language (standardized score)

b: Non-verbal intelligence (standardized score)

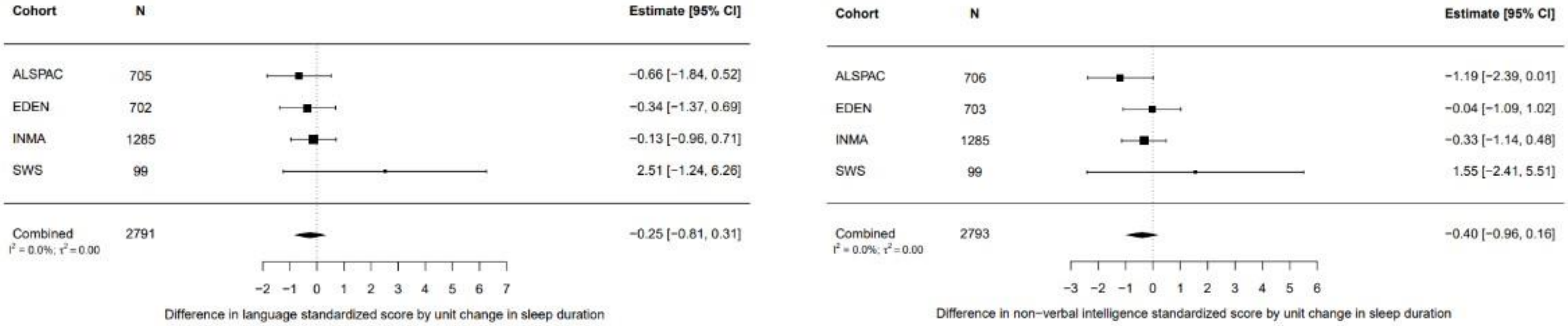

Adjusted for sex of the child, age at outcome measurement, cohort, mother's age at birth, maternal education, mother born abroad, birthweight, gestational age, sibling position, smoking during pregnancy, EUSILC-based household income

In the ALSPAC cohort twins were included, in the EDEN, INMA and SWS cohort twins were an exclusion criteria. In the EDEN and SWS cohort children with congenital anomalies were included, in the ALSPAC and INMA cohort it was an exclusion criteria.

Abbreviations: ALSPAC: Avon Longitudinal Study of Parents and Children, CI: Confidence interval, EDEN: Étude des Déterminants pré et postnatals du développement et de la santé de l'Enfant, INMA: INfancia y Medio Ambiente Project, SWS: Southampton Women's Survey, N: Number of children included in the analysis; I^2 and τ^2 statistics represent between cohort heterogeneity

Figure 13: Association between total sleep duration per day at mean age of 3.7 years and 13a) language (standardized score), 13b) non-verbal intelligence (standardized score) at mean age of 4.9 years using two-stage IPD meta-analysis: INMA excluded from analysis – adjusted models

a: Language (standardized score)

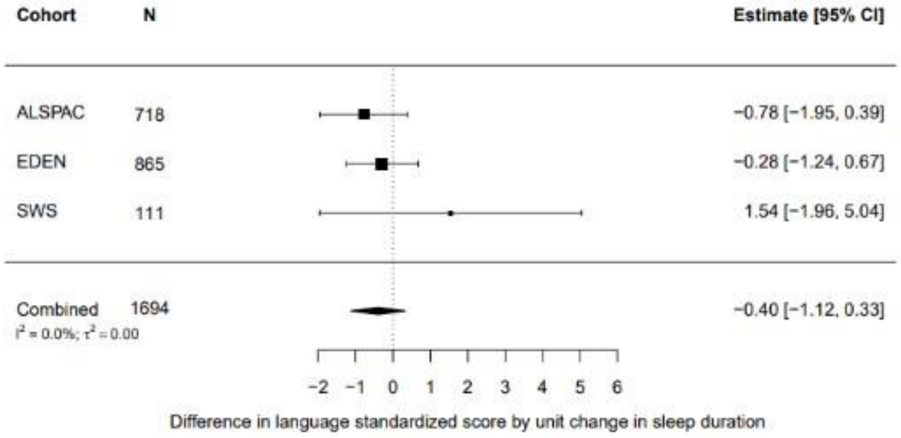

b: Non-verbal intelligence (standardized score)

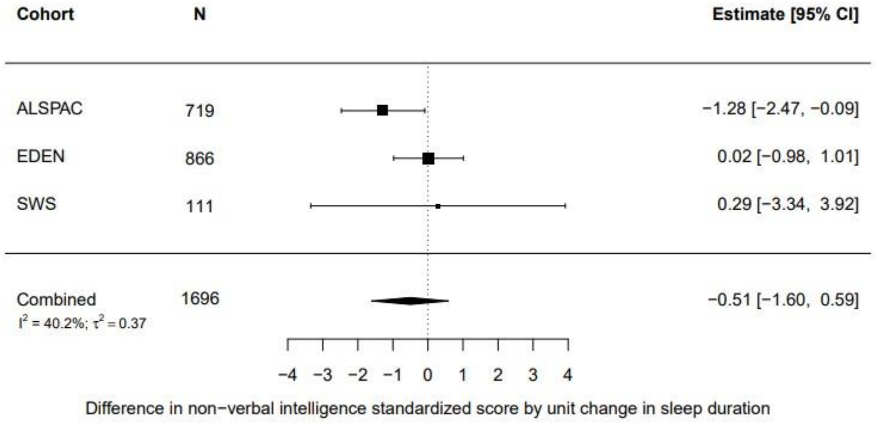

Adjusted for sex of the child, age at outcome measurement, cohort, mother's age at birth, maternal education, mother born abroad, birthweight, gestational age, sibling position, smoking during pregnancy, EUSILC-based household income

Abbreviations: ALSPAC: Avon Longitudinal Study of Parents and Children, CI: Confidence interval, EDEN: Étude des Déterminants pré et postnatals du développement et de la santé de l'Enfant, INMA: INfancia y Medio Ambiente Project, SWS: Southampton Women's Survey, N: Number of children included in the analysis; I^2 and τ^2 statistics represent between cohort heterogeneity
